# Paxillin Mediates Sensing of Physical Cues and Regulates Directional Cell Motility by Controlling Lamellipodia Positioning

**DOI:** 10.1371/journal.pone.0028303

**Published:** 2011-12-14

**Authors:** Julia E. Sero, Charles K. Thodeti, Akiko Mammoto, Chris Bakal, Sheila Thomas, Donald E. Ingber

**Affiliations:** 1 Vascular Biology Program, Departments of Pathology and Surgery, Children's Hospital Boston and Harvard Medical School, Boston, Massachusetts, United States of America; 2 Beth Israel Deaconess Medical Center and Harvard Medical School, Boston, Massachusetts, United States of America; 3 Wyss Institute for Biologically Inspired Engineering at Harvard University, Boston, Massachusetts, United States of America; 4 School of Engineering and Applied Sciences, Harvard University, Cambridge, Massachusetts, United States of America; 5 Dynamical Cell Systems Team, Division of Cancer Biology, Institute of Cancer Research, London, United Kingdom; King's College London, United Kingdom

## Abstract

Physical interactions between cells and the extracellular matrix (ECM) guide directional migration by spatially controlling where cells form focal adhesions (FAs), which in turn regulate the extension of motile processes. Here we show that physical control of directional migration requires the FA scaffold protein paxillin. Using single-cell sized ECM islands to constrain cell shape, we found that fibroblasts cultured on square islands preferentially activated Rac and extended lamellipodia from corner, rather than side regions after 30 min stimulation with PDGF, but that cells lacking paxillin failed to restrict Rac activity to corners and formed small lamellipodia along their entire peripheries. This spatial preference was preceded by non-spatially constrained formation of both dorsal and lateral membrane ruffles from 5–10 min. Expression of paxillin N-terminal (paxN) or C-terminal (paxC) truncation mutants produced opposite, but complementary, effects on lamellipodia formation. Surprisingly, pax−/− and paxN cells also formed more circular dorsal ruffles (CDRs) than pax+ cells, while paxC cells formed fewer CDRs and extended larger lamellipodia even in the absence of PDGF. In a two-dimensional (2D) wound assay, pax−/− cells migrated at similar speeds to controls but lost directional persistence. Directional motility was rescued by expressing full-length paxillin or the N-terminus alone, but paxN cells migrated more slowly. In contrast, pax−/− and paxN cells exhibited increased migration in a three-dimensional (3D) invasion assay, with paxN cells invading Matrigel even in the absence of PDGF. These studies indicate that paxillin integrates physical and chemical motility signals by spatially constraining where cells will form motile processes, and thereby regulates directional migration both in 2D and 3D. These findings also suggest that CDRs may correspond to invasive protrusions that drive cell migration through 3D extracellular matrices.

## Introduction

Directional cell migration is a multi-step process that involves actin-driven protrusion of the plasma membrane, designation of a leading edge, formation of new cell-extracellular matrix (ECM) adhesions, contraction of the cytoskeleton, and disassembly of rearward adhesions [1]. Although many studies have focused on migration directed by gradients of soluble factors, directional motility also can be physically controlled by adhesive gradients (haptotaxis [2]), mechanical stiffness (durotaxis [3], [4]); alignment of ECM features (contact guidance [5], [6], [7]), and variations in the geometry of the ECM that affect cell shape (shape-dependent motility control [8], [9], [10], [11]).

Cell spreading on adhesive substrates is driven in part by cytoskeletal traction forces that are resisted mechanically by the ECM [12], [13]. Mechanical forces are transmitted between the ECM and cytoskeleton through transmembrane receptors, such as integrins, which are coupled to the cytoskeleton via adaptor proteins in multi-protein anchoring complexes called focal adhesions (FAs) [14]. FAs also function as platforms for signal transduction, as they include many signaling molecules as well as load-bearing scaffold proteins [15], [16], [17]. Thus, FAs are now considered to be mechanosensitive organelles that facilitate the conversion of mechanical and spatial cues from the microenvironment into changes in cytoskeletal architecture and biochemical signaling [12], [15], [17].

Physical interactions between a cell and the ECM can direct migration by guiding where the cell extends new motile processes, such as lamellipodia and filipodia [8], [9], [18], [19], [20]. Cytokine-induced activation of the small GTPase Rac and actin-driven membrane protrusion have been reported to occur in close proximity to FAs in several cell types [8], [9], [11], [21]. Furthermore, directional migration can be directly controlled by artificially positioning FAs using micropatterned adhesive substrates [11]. However, the molecular mechanism by which FA position is spatially coupled to Rac activation and lamellipodia extension remains unclear.

The FA protein paxillin associates with many signaling proteins, including FAK [22] and other kinases, protein phosphatases, and small GTPase activators and effectors [23], as well as structural proteins such as vinculin [24]. Paxillin-null mouse embryonic fibroblasts (MEFs) and embryonic stem cells also have defects in spreading and migration, FA remodeling, and forming stable lamellipodia [25], [26]. Moreover, paxillin mutations have been implicated in the poor prognosis of various invasive tumors, including breast [27], [28], lung [29], [30], and melanoma [31], [32], suggesting that paxillin is important for controlling cell migration and invasion in living tissues. Thus, in the present study, we set out to test whether paxillin is required for spatially coupling lamellipodia formation to sites of cell-ECM attachment.

To investigate whether paxillin is required for directional lamellipodia extension, we cultured cells on square-shaped, cell-sized adhesive ECM islands fabricated by microcontact printing. We previously showed that cells plated on similar square ECM islands consistently form FAs in their corners, where cell distortion and traction forces are highest, and that they extend motile processes from corner regions when stimulated with PDGF [9]. Here, we leveraged this ability to predict where new lamellipodia will form to dissect out the role of paxillin in guiding directional cell migration by studying paxillin knockouts and cells expressing paxillin truncation mutants. In the course of these studies, we made the unexpected observation that paxillin-null fibroblasts had a higher propensity to form circular dorsal ruffles (CDRs) when stimulated with PDGF. Because CDRs have been proposed to function as invasive motile structures [53], we extended this work to analyze the role of paxillin in directional migration in 3D matrices.

## Results

### Focal adhesions and lamellipodia are spatially restricted in square cells

**Figure 1:**
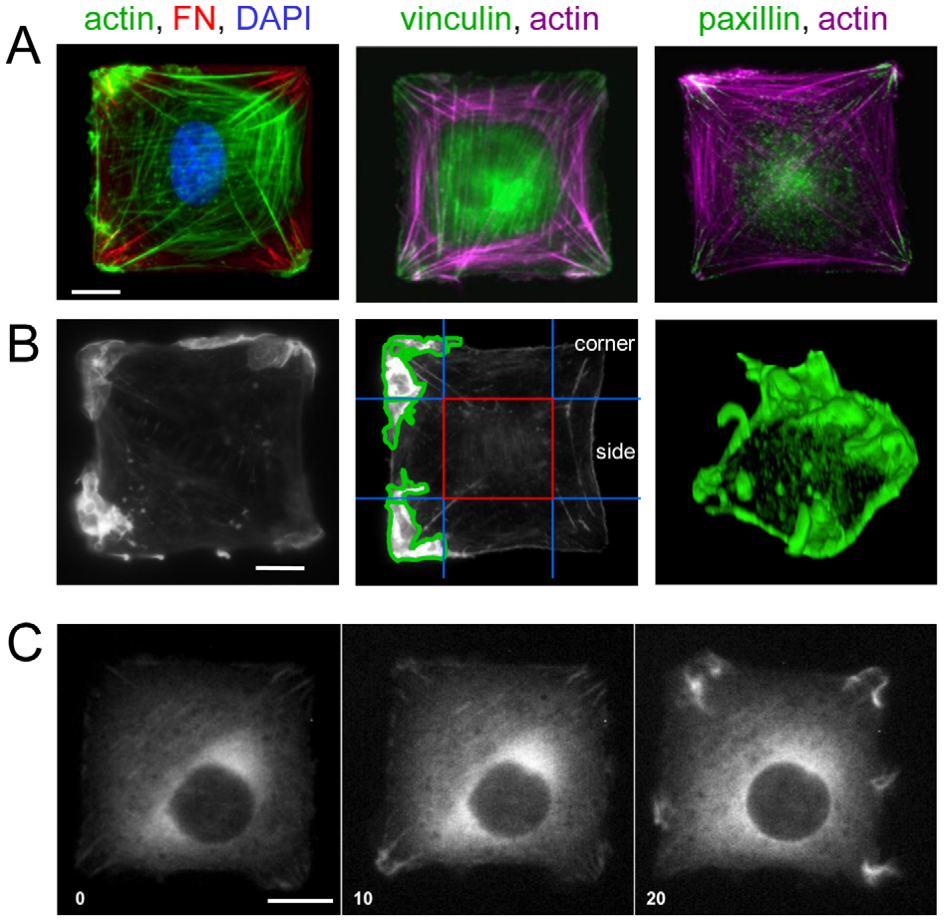
Cells on microcontact-printed square islands form focal adhesions (FAs) and lamellipodia preferentially in corner regions. A) Primary human dermal (left) and lung (middle and right) fibroblasts align actin stress fibers (left, green; middle and right, magenta) along diagonal axes and form fibronectin (FN) fibrils (left, red) and FAs containing vinculin (middle, green) and paxillin (right, green). B) Cells stimulated with PDGF (25 ng/mL) for 30 min and stained with Alexa488-phalloidin show actin-rich protrusive structures predominantly from corner regions, defined as shown (middle). 3D reconstruction of confocal sections (right) shows that lamellipodia could fold back over cell bodies, due to a lack of adhesive substrate surrounding the adhesive islands. C) Time-lapse microscopy of GFP-paxillin in an NIH 3T3 cell stimulated with PDGF (25 ng/mL) shows large FAs that disassemble over time and paxillin-containing protrusions emanating largely from corners. Time = min after addition of PDGF. Scale bars = 10 µm.

Microcontact-printed substrates consisting of arrays of square ECM islands (900–2500 µm^2^) surrounded by non-adhesive regions were prepared by direct stamping of fibronectin (FN) onto activated PDMS-coated coverslips, followed by blocking of unstamped areas with Pluronic F-127 (Fig. S1) [33], [34]. Human fibroblasts plated on these islands spread and adopted square shapes, as previously reported for cells cultured on ECM islands formed by stamping self-assembled monolayers of alkanethiols on gold [9]. These square cells formed actin stress fibers aligned primarily along their diagonal axes (Fig. 1A, left) that terminated in corner-localized FAs, which contained vinculin (Fig. 1A, middle) and paxillin (Fig. 1A, right). Cells also formed extracellular FN fibrils beneath the FAs in their corner regions where cell traction forces are concentrated [9], [35] (Fig. 1A, left). Thus, fibroblasts on square-shaped islands are “artificially polarized” in response to the geometry of the adhesive ECM. Consistent with previous findings [9], square-shaped human fibroblasts and mouse embryonic fibroblasts (MEFs) both extended large actin-containing membrane protrusions predominantly from their corner regions when analyzed 30 min after stimulation with PDGF (25 ng/ml) (Fig. 1B, left and middle). 3D reconstruction of confocal sections revealed that these structures often extended upwards from the cell periphery and folded back over the cell body, likely because the surrounding substrate was non-adhesive (Fig. 1B, right).

To analyze the spatiotemporal dynamics of FAs during lamellipodia formation, we performed time-lapse microscopy on wild-type 3T3 fibroblasts that were transiently transfected with GFP-paxillin and cultured on square FN islands in serum-free medium. Prior to addition of PDGF, GFP-paxillin was present in large corner FAs (Fig. 1C, left) whereas at 10 min after PDGF stimulation, a portion of the GFP-paxillin translocated centripetally into adjacent membrane ruffles that extended outward from FAs over the nearby corners of the cell (Fig. 1C, middle). This was accompanied by the shrinkage and disappearance of other FAs, indicating that PDGF also stimulated FA turnover. This observation confirms that paxillin is positioned at sites where it could directly link physical signals to membrane extension.

### Loss of paxillin leads to impaired spatial control of lamellipodia formation

**Figure 2:**
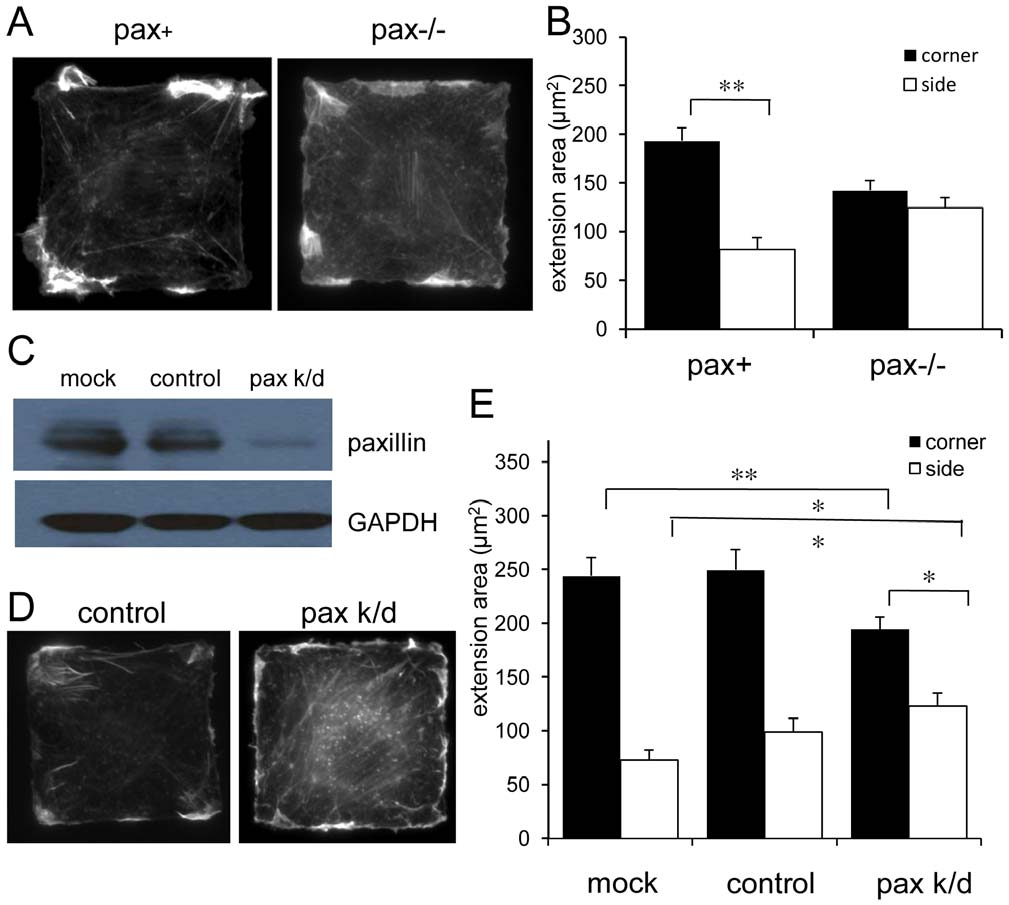
Paxillin-deficient cells do not show corner preference for lamellipodia formation. A) Paxillin −/− and pax+ (rescued) mouse embryonic fibroblasts (MEFs) plated on 50×50 µm square FN islands stimulated with PDGF (25 ng/ml) for 30 min, fixed and stained with Alexa488-phalloidin to label F-actin. B) Quantification of corner and side membrane extension area in pax+ and pax−/− MEFs (as shown in Fig. 1B). n>100 cells per genotype. ** *p*<0.001. C) siRNA-mediated knockdown of paxillin in human lung fibroblasts (IMR-90) resulted in almost complete loss of paxillin expression as shown by Western blot. D) IMR-90 cells transfected with negative control or siRNA against human paxillin plated on 50×50 µm islands stimulated with PDGF (25 ng/ml) for 30 min, stained with Alexa488-phalloidin. E) Quantification of corner and side extension area in mock-, control-, and paxillin-siRNA transfected cells. n>30 cells per genotype. ** *p*<0.005. * *p*<0.01.

In order to determine whether paxillin is required for coupling FA position and lamellipodia extension, we tested the ability of paxillin-deficient cells to form lamellipodia on square ECM islands. We initially studied a pax−/− MEF (pax−/−) cell line that was previously derived from E7.5 paxillin knockout mouse embryos; as controls, we used the same knockout MEFs that were engineered using retroviral infection to stably express myc-tagged mouse paxillin (pax+) [36]. Both pax+ and pax−/− cells spread on square FN islands and extended new lamellipodia after 30 min of PDGF stimulation (Fig. 2A). The total area of newly extended membrane per cell did not differ significantly; however, only pax+ cells showed a strong preference to form lamellipodia in the corners of these cells (*p*<0.001), as quantified by computerized image analysis (Fig. 2B).

To confirm that the inability of pax−/− cells to spatially restrict lamellipodia to corners was specifically due to loss of paxillin, we used siRNA to selectively knock down paxillin expression in normal human lung IMR-90 fibroblasts (IMR-90) [37]. Paxillin protein levels were reduced by ∼90% in the knockdown (pax k/d) cells compared to control siRNA- and mock-transfected cells at 72 h post transfection (Fig. 2C). Pax k/d cells (Fig. 2D) also displayed significantly lower corner extension area (*p*<0.005), and higher side extension area (*p*<0.005) compared to control cells (Fig. 2E), and similar results were obtained using primary human dermal fibroblasts (HDFs) (data not shown). The human pax k/d cells retained a small corner lamellipodia preference (*p*<0.01), which may be due to residual paxillin expression, the presence of untransfected cells, or differences between the cell types.

**Figure 3:**
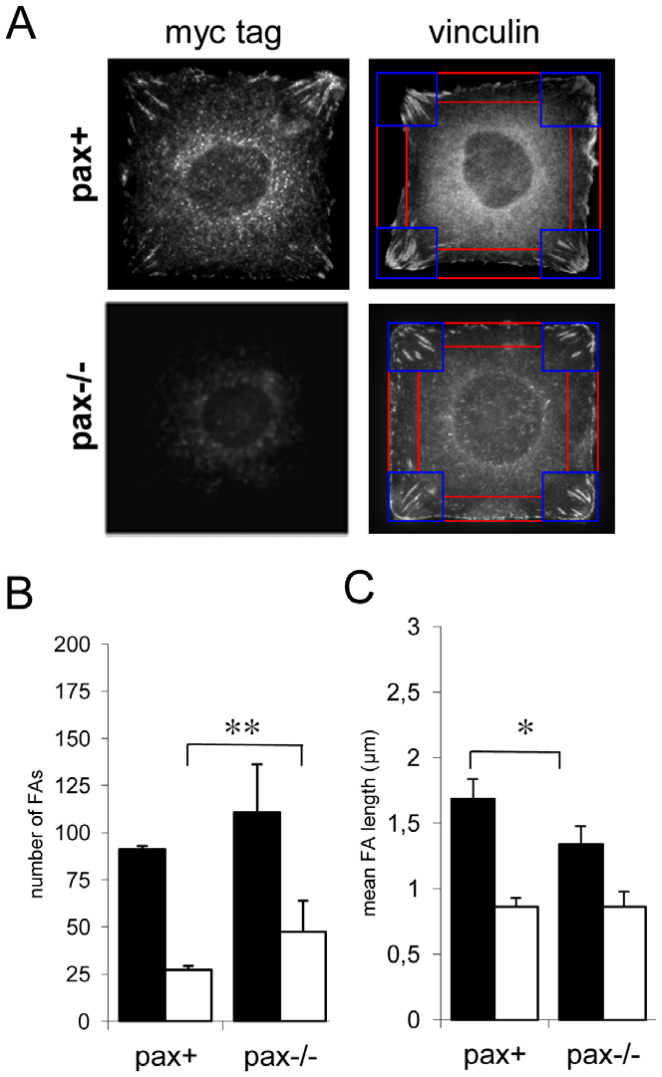
The number and distribution of vinculin-containing adhesive complexes is abnormal in paxillin knockout cells. A) Myc-tagged paxillin is localized to predominantly corner-localized FAs in pax+, but not pax−/−, cells (left). Both pax+ and pax−/− cells show FAs as detected by immunostaining for vinculin (right), but pax−/− cells have more adhesive structures in side regions (red) and fewer long, fibrillar type adhesions in corner regions (blue). B) Quantification of number of FAs in corner (black bars) and side regions (white bars). n>6 cells, 400 FAs per genotype. ** *p*<0.005. C) Quantification of FA length in corner (black bars) and side regions (white bars). * *p*<0.05.

We next quantified FAs in square cells to determine whether paxillin loss affects the positioning of adhesive complexes. Both pax+ and pax−/− MEFs formed corner-localized vinculin-containing FAs (Fig. 3A, right), but pax−/− cells had more FAs in side regions than pax+ cells (*p*<0.005) (Fig. 3B) and the average length of corner FAs was shorter (*p*<0.05) (Fig. 3C). A similar increase in the number of side FAs was observed for IMR-90 cells treated with control or paxillin siRNA (*p*<0.05) (data not shown). These findings support the hypothesis that FA position correlates spatially with the position of new membrane process formation, and further implicate paxillin as a key regulator of positioning of both FAs and lamellipodia inside the cell.

### Paxillin is required for spatial restriction of Rac activity

**Figure 4:**
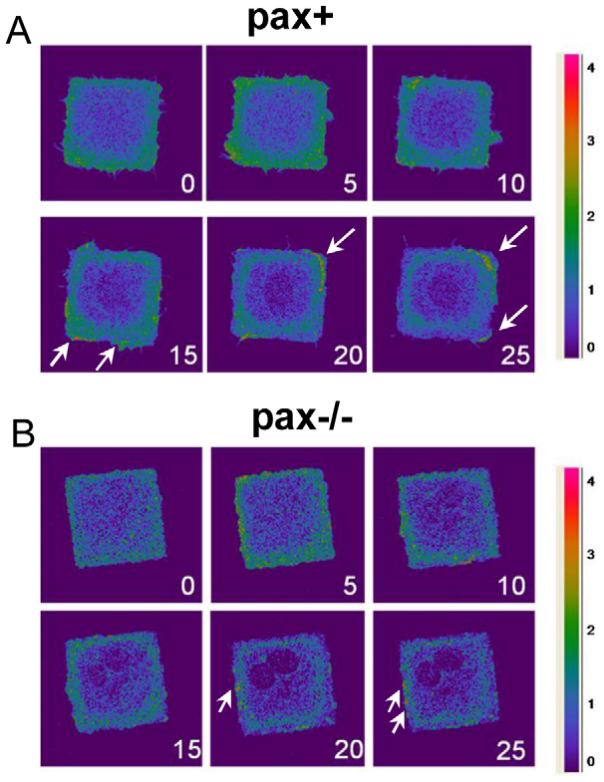
Rac activation is localized to corner regions in control, but not paxillin-deficient, square cells. Pax+ (A) and pax−/− (B) MEFs were transfected with a Raichu-Rac FRET construct, plated on 50×50 µm FN islands, and stimulated with PDGF (25 ng/ml). FRET, indicating Rac activation, was imaged every 60 s using laser confocal microscopy. Arrows indicate areas with high FRET (green-red), i.e. local Rac activation.

Lamellipodia formation is driven by Rac activation [38], which can occur at or near FAs [11]. Paxillin acts as a scaffold protein for various Rac signaling complexes such as CrkII/DOCK180/ELMO [39], [40] and Pkl-PIX-PAK [41]. Thus, the loss of directional lamellipodia extension in square pax−/− cells raised the possibility that paxillin couples Rac activation to FA position. We therefore investigated the spatiotemporal dynamics of Rac activation in live square cells using the Raichu-Rac FRET probe as a readout of Rac GEF activity [42]. Rac activation increased in the first 5 min after addition of PDGF without spatial preference in both pax+ (Fig. 4A) and pax−/− (Fig. 4B) cells. From 15 through 25 min, however, high levels of Rac were predominantly localized to corner regions much like the pattern of lamellipodia observed in pax+ cells (Fig. 4A, arrows). In contrast, Rac activity was distributed in a more punctate pattern in pax−/− MEFs and it was not constrained to corner regions (Fig. 4B, arrows), consistent with the small, non-localized lamellipodia we observed in these cells (Fig. 2).

### Paxillin is required for normal spatiotemporal dynamics of lamellipodia extension

**Figure 5:**
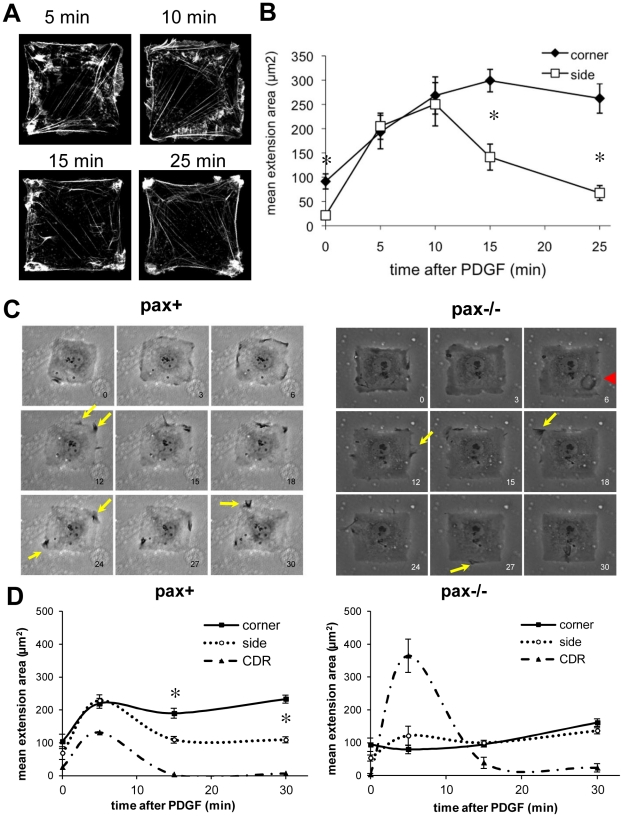
Paxillin is involved in coordinating the spatiotemporal dynamics of membrane extension in response to PDGF stimulation in square cells. The early motile response to PDGF consists of an initial unconstrained phase of dorsal and lateral protrusion (3–10 min) followed by progressive spatial restriction of new process formation (15–30 min). A) Human dermal fibroblasts plated on 50×50 µm FN islands were treated with PDGF (25 ng/ml) for the indicated times, fixed, and stained with Alexa488-phalloidin to label F-actin. Both dorsal and lateral protrusions are visible at 5 and 10 min, and by 15 min, lateral lamellipodia are largely constrained to corners. B) Membrane extension areas were measured in corner and side regions at each time-point. n>25 cells per time-point. * *p*<0.01 between corner and side extension areas. C) Time-lapse video microscopy of pax+ (left) and pax−/− (right) cells on 50×50 µm FN islands shows initial ruffling from the cell peripheries as well as the formation of a circular dorsal ruffle (CDR) in the pax−/− cell at 6 min (red arrowhead). After 10 min, at least two rounds of membrane extension occur and are progressively constrained to corners in the pax+ cell, but not the pax−/− cell (yellow arrows). D) Quantification of corner, side, and CDR areas in cells fixed at 0, 5, 15, and 30 min after PDGF stimulation in pax+ (left) and pax−/− (right) cells. n>10 cells per time-point. * *p*<0.01 between corner and side areas.

These data suggest that paxillin is involved in both promoting Rac-based lamellipodia formation near FAs in corner regions and suppressing membrane extension at the sides of our artificially polarized square cells. We next examined the time-course of lamellipodia formation to further elucidate the role of paxillin in control of directional membrane extension. Wild-type HDFs stimulated with PDGF formed extensive protrusions around the entire periphery of the cell by 5 to 10 min; however, lamellipodia became limited primarily to the corners by 15 min and they were almost entirely restricted to corner regions by 30 min (Fig. 5A). Quantification using computerized image analysis confirmed that there was an initial early burst of membrane extension without spatial constraint from 5 to 10 min after addition of PDGF, followed by progressive restriction of lamellipodia to corners (Fig. 5B), which corresponds to the dynamics of Rac activation observed in pax+ cells expressing the Raichu-Rac FRET probe (Fig. 4). Interestingly, protrusive activity was not limited to lateral edges of cells, as bright, actin-rich, dorsal ruffling protrusions also were detected at 5 and 10 min, though not at later times (Fig. 5A).

We also examined the dynamics of lamellipodia formation in living pax+ and pax−/− MEFs using phase-contrast microscopy. By 3 min after addition of PDGF, extensive membrane ruffles formed along all of the edges of a square pax+ cell, which then folded back over the cell body by 6 min (Fig. 5C left). Again, subsequent rounds of membrane extension were progressively constrained to corner regions, such that fan-shaped lamellipodia formed almost exclusively from corners by 24 min. An initial burst of lateral membrane ruffling was also observed by 3 min in pax−/− cells (Fig. 5C, right). However, large CDRs also formed on their apical surfaces by 6 min, which then contracted and were internalized within phase-light, micropinosome-like structures [42]. Fan-shaped lamellipodia and whiskery filopodium-like structures continued to form both along the sides and in corner regions for at least 30 min in these pax −/− cells (Fig. 5C).

More careful detailed quantification of these results in pax+ and pax−/− cells fixed at various time points confirmed that square pax+ cells formed lamellipodia with no spatial preference as well as CDRs at 5 min (Fig. 5D). This was followed by a decrease in the area of side lamellipodia, so that cells showed a significant corner lamellipodia preference and virtually all CDRs disappeared by 15 min (Figs. 5D). Pax−/− cells, on the other hand, formed significantly larger CDRs and smaller lateral lamellipodia than pax+ cells at 5 min (Figs. 5D). The lamellipodia then increased equally in corners and sides from 15 through 30 min, and some CDRs were still present at 30 min (Fig. 5D, right). These observations confirm that cells respond to PDGF by extending protrusions in an unconstrained manner at early times (3–10 min), but in pax+ cells this is then followed by successive rounds of spatially-restricted membrane extension, consistent with previously reported observations [9]. Moreover, paxillin appears to be required for both suppressing side lamellipodia and promoting corner lamellipodia during the latter part of this response. Surprisingly, paxillin also seems to have an important function outside of FAs in that it also regulates formation of CDR protrusions on the apical membrane.

### The N- and C-termini of paxillin control membrane extension in opposing ways

**Figure 6:**
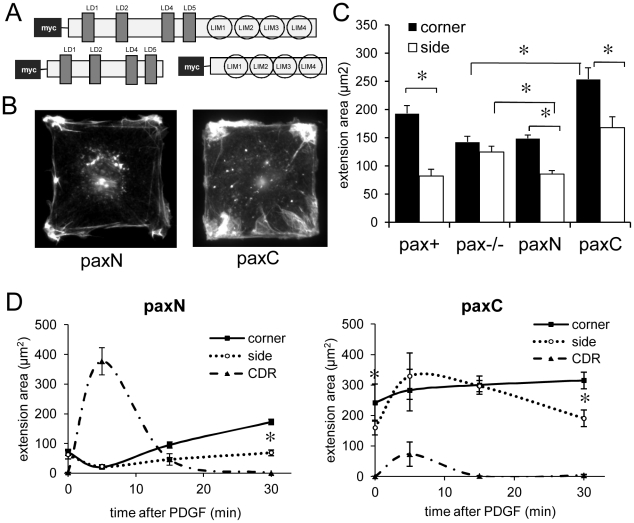
Expression of the N-terminal half of paxillin leads to suppression of side extensions and broad lamellipodia, whereas the C-terminus alone enhances lamellipodia formation. A) Schematic of the structure of the myc-tagged paxillin constructs expressed in pax+, paxN, and paxC MEFs. B) PaxN (left) and paxC (right) MEFs plated on 50×50 µm FN islands were treated with PDGF (25 ng/ml) for the indicated times, fixed, and stained with Alexa488-phalloidin to label F-actin. C) Quantification of corner and side extension areas in cells fixed 30 min after PDGF stimulation. Both paxN and paxC expression result in significant differences between corner and side extension areas. Side extension area is significantly reduced in paxN Cells compared to pax−/− cells, and corner extension area is enhanced in paxC cells compared to pax−/− cells. n>50 cells per genotype. * *p*<0.001. D) Spatiotemporal dynamics of membrane extension in paxN and paxC cells fixed at 0, 5, 15, and 30 min after addition of PDGF. PaxN cells (left) mainly form CDRs at 5 min, then extend small corner-localized protrusions by 30 min. PaxC cells (right) form corner lamellipodia even in the absence of PDGF, which induces transient lateral membrane extension. n>20 cells per genotype. * *p*<0.01 between corner and side extension areas.

We next explored the molecular mechanism by which paxillin regulates formation of lamellipodia and CDRs by testing the ability of stably-expressed, myc-tagged truncation mutants comprising either the N-terminus (paxN) or C-terminus (paxC) of mouse paxillin to rescue the corner lamellipodia preference in square pax −/− MEFs (Fig. 6A). The paxillin N-terminus contains several short alpha-helical motifs that bind other FA proteins and signaling molecules, such as vinculin [24], FAK and Src kinases [43] and the Arf6GAP PKL [41], as well as a polyproline domain and long regions of random coil [44]. The C-terminus is composed of four tandem LIM zinc finger domains, and it contains the FA-targeting sequence [45], as well as binding sites for tubulin [46] and and PTP-PEST [47], [48], [49]. Importantly, calpain-mediated proteolysis of paxillin regulates lamellipodia formation [50] and FA dynamics [51], suggesting that the N- and C-termini might also have distinct effects on cell physiology.

Expression of either paxN or paxC alone was sufficient to restore a corner lamellipodia preference in PDGF-stimulated pax −/− cells, but through apparently opposite mechanisms. Expression of paxN rescued the suppression of side protrusions (*p*<0.01), but it did not lead to an increase in corner lamellipodia area (Fig. 6B, C). The total area of membrane extension was also lower in paxN cells than in either pax+ or pax−/− cells (*p*<0.01). Expression of paxC, on the other hand, resulted in a dramatic increase in total membrane extension area (*p*<0.001), as well as restoration of the corner lamellipodia preference (Fig. 6B, C). However, this was due to the nearly two-fold increase in corner extension area compared to pax−/− cells (*p*<0.001), rather than to suppression of lamellipodia formation at the sides (Fig. 6C). The morphologies of the truncation mutant membrane extensions were also distinct. PDGF-stimulated paxN cells formed whiskery, filopodium-like extensions that emanated from tight foci and remained bundled, rather than forming broad waves; these also were even more tightly corner-localized than in pax+ cells (Fig. 6B left). In contrast, paxC cells formed large, fan-shaped lamellipodia from both corners and sides (Fig. 6B, right).

Next, we characterized the spatiotemporal dynamics of membrane extension in paxN and paxC cells in response to PDGF stimulation. Interestingly, paxN cells extended few lateral membrane processes at 5 or even 15 min after PDGF addition, although they did form extensive CDRs (Fig. 6D and Fig. S2A). However, by 30 min, only small membrane protrusions remained that were predominantly in corner regions (Fig. 6C, D). Surprisingly, paxC cells formed lamellipodia even in the absence of growth factors, and they displayed a marked corner preference from the start of the experiment (Fig. 6D). At 5 min after PDGF stimulation, side lamellipodia formation increased transiently due to a burst of peripheral ruffling (*p* = 0.03), whereas corner lamellipodia area increased only slightly from the high baseline value, and few cells formed CDRs (Fig. 6D and Fig. S2A). By 30 min, side extension areas returned to pre-stimulation levels (which were significantly greater than those of pax+ and pax−/− cells) and large corner lamellipodia remained (Fig. 6D).

Indirect immunostaining of the myc tag in paxN cells (Fig. S3A) and fluorescence microscopy studies using transiently expressed GFP-paxN protein (Fig. S4A) revealed that the N-terminus of paxillin was not highly enriched in FAs, even though it contains binding sites for many FA proteins; instead, it was predominantly located in the cytoplasm and perinuclear regions. Consistent with the reported presence of the FA targeting domain in the C-terminus [45], both myc-paxC (Fig. S3A) and GFP-paxC (Fig. S4A) were detectable in FAs in addition to being highly expressed throughout the cytosol and nucleus. PaxN cells also formed more and longer FAs than pax+ cells, particularly in corner regions, (Fig. S3B), whereas paxC cells formed more side FAs and shorter corner adhesions (Fig. S3C). The expression level of paxN protein also was consistently lower than that of the full-length or paxC proteins when analyzed by immunostaining or Western blots (Fig. S4C); this might be due to degradation of the cytosolic paxN fragment.

### Co-expression of paxN and paxC rescues spatial control of lamellipodia formation

Because paxN and paxC appeared to exert opposing effects on membrane extension, we tested the mutants in combination with one another, or with full-length paxillin, in order to determine whether the N- or C-termini had dominant effects in pax+ cells, as well as whether the two separate halves of the protein could exert complementary effects in the same cell. To do this, we expressed GFP-tagged avian paxillin constructs in pax−/−, pax+, paxN, and paxC cells by transient transfection.

**Figure 7:**
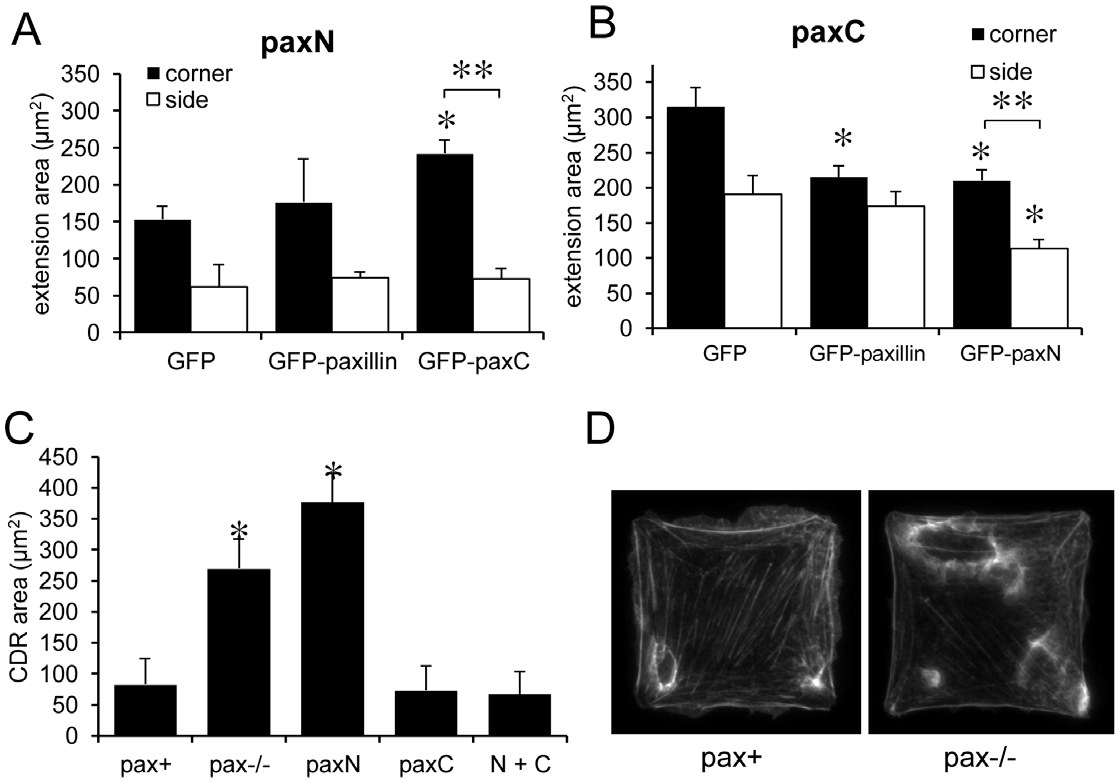
PaxN and paxC exert complementary effects on lamellipodia formation, and co-expression of the separate halves of paxillin can rescue both corner lamellipodia formation and suppression of CDRs and side membrane extension. A) GFP, GFP-paxillin, or GFP-paxC were transiently expressed in paxN MEFs plated on 50×50 µm FN islands, stimulated with PDGF (25 ng/ml) for 30 min, fixed and stained to label F-actin. Expression of GFP-paxC, but not GFP alone or GFP-paxillin, could rescue enhancement of corner lamellipodia formation. B) GFP, GFP-paxillin, or GFP-paxN were transiently expressed in paxC MEFs as in A. Expression of GFP-paxillin and GFP-paxN suppressed corner extension area, but only expression of GFP-paxN also suppressed side extension area. n>15 cells per genotype. * *p*<0.01 compared to GFP alone. ** p<0.001 between corner and side extension areas. C) Quantification of CDR area in square cells stimulated with PDGF for 5 min shows that paxC rescues CDR suppression when expressed in either pax−/− or paxN cells. * *p*<0.01 compared to pax+. D) Pax+ and pax−/− cells fixed at 5 min and stained to label for F-actin to visualize CDRs.

Expression of intact GFP-paxillin was sufficient to rescue the corner lamellipodia preference in pax−/− cells (*p*<0.01), with both increased corner extension area and decreased side extension area compared to GFP alone (Fig. S4B). Transient expression of GFP-paxN or GFP-paxC each had similar effects on membrane extension as the stably expressed myc-tagged mutants (Fig. S4B). After 30 min of PDGF stimulation, paxN cells expressing GFP-paxillin did not show a statistically significant increase in corner membrane extension area compared to cells transfected with GFP (Fig. 7A). PaxN cells transfected with GFP-paxC, however, formed significantly more membrane extensions in corners (*p*<0.01) (Fig. 7A). In fact, both corner and side extension areas in paxN cells expressing GFP-paxC were comparable to those of cells expressing full-length GFP-paxillin. Expression of GFP-paxillin in paxC cells significantly reduced corner lamellipodia area (*p*<0.01), but did not rescue suppression of side lamellipodia (Fig. 7B). Co-expression of GFP-paxN with paxC, however, did lead to suppression of side lamellipodia, and thus rescued the corner lamellipodia preference (*p*<0.001). Again, both corner and side extension areas in these cells were comparable to those of cells expressing full-length GFP-paxillin.

We had observed that pax −/−cells have a greater propensity to form CDRs on their apical membranes than pax+ cells (Fig. 7C,D). Interestingly, expression of GFP-paxC in pax −/− cells suppressed CDR formation, where as expression of GFP-paxN had no significant effect (Fig. 7C). Moreover, expression of GFP-paxN in paxC cells did not result in an increase in CDR formation (Fig. 7C), which implies that the paxillin C-terminus may actively suppress dorsal ruffling as well as promote lateral protrusion.

### Paxillin differentially regulates dorsal ruffling through its N- and C-termini

**Figure 8:**
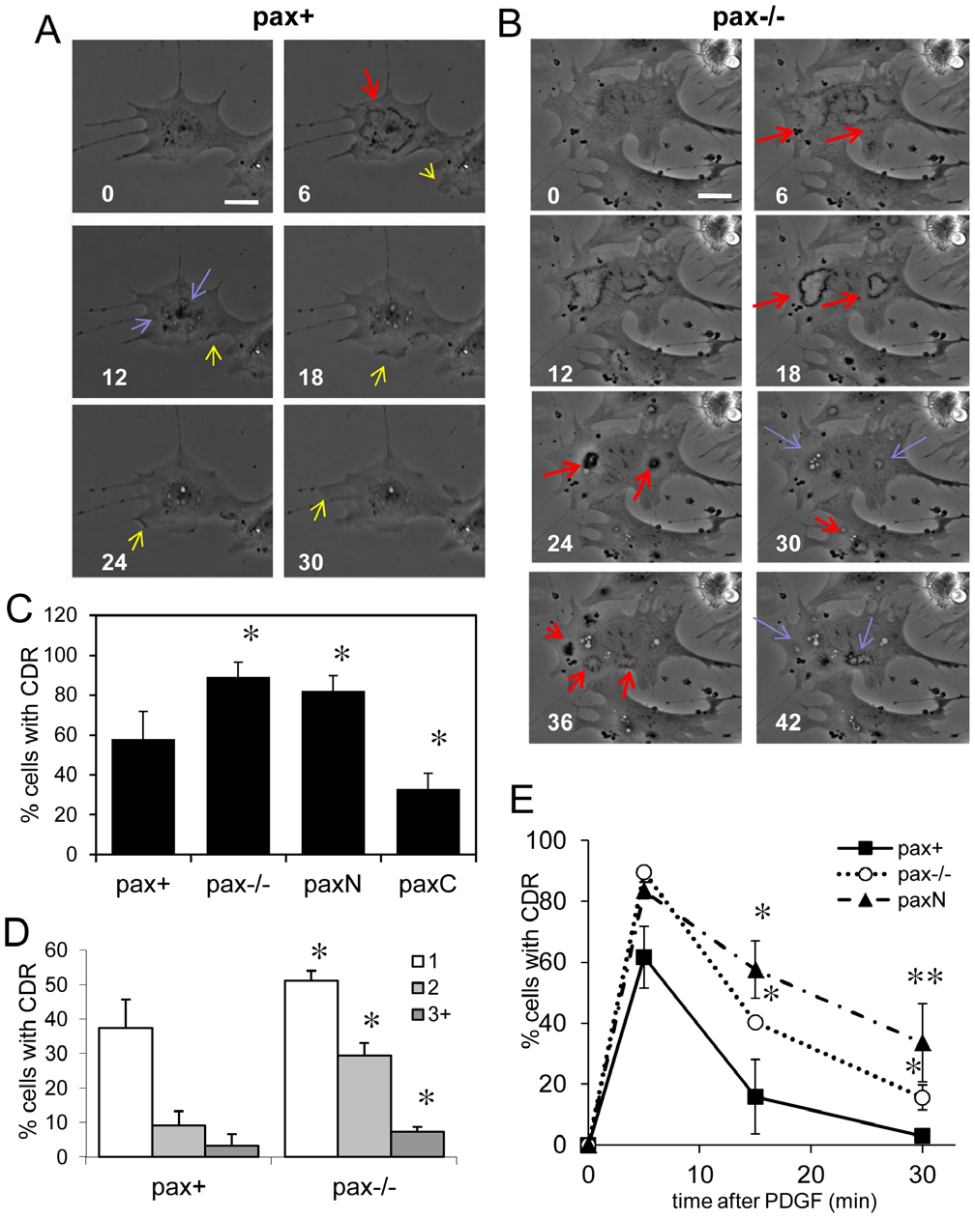
Pax−/− and paxN cells form more CDRs than pax+ and paxC cells and undergo repeated rounds of CDR formation in response of PDGF. Live cells were imaged before and after addition of PDGF (25 ng/ml) by phase-contrast time-lapse microscopy. A) Pax+ cell shows formation of a CDR (red arrow) and lateral membrane extension (yellow arrow). By 12 min, the CDR has been completely internalized into macropinosomal structures (blue arrows). Lateral ruffling continues and no further CDRs form through 30 min (and beyond). B) Pax−/− cells form several CDRs per cell which persist through 18–24 min before being completely internalized (red arrows) into multiple macripinosomes that remain in the cytosol (blue arrows). Multiple rounds of CDR formation take place in the same cells at 30 and 36 min. (Similar dynamics were observed in paxN cells by time-lapse.) Scale bar = 10 µm. C) Percent of CDR-positive pax+, pax−/−, paxN, and paxC cells grown on unpatterned FN stimulated with PDGF for 5 min, fixed, and stained for F-actin. n>200 cells per genotype. * *p*<0.01 compared to pax+. D) Percent of unpatterned cells with 1, 2, and 3 or more CDR at 5 min. * *p*<0.01 compared to pax+. E) Quantification of percent of cells with CDR from time-lapse movies of cells pax+, pax−/−, and paxN cells stimulated with PDGF. n>40 cells per genotype. * *p*<0.01. ** *p*<0.001.

The experiments described so far were performed with cells cultured on microengineered square ECM islands, so we next tested the effects of paxillin mutation on cells cultured on standard 2D culture substrates to rule out the possibility that the effects we observed were an artifact of this model system. Time-lapse microscopy confirmed that pax+ cells formed both lateral lamellipodia and CDRs in response to PDGF stimulation at early times (Fig. 8A). These cells typically underwent a single round of CDR formation, which was completed within 10 min (Fig. 8A, E), with most dorsal ruffles being completely internalized by 15 min, after which extensive lateral lamellipodia formation continued through 30 min and beyond. Phase-lucent vesicles formed beneath the sites of CDR internalization, then translocated toward the nucleus and decreased in size by 30 min. In contrast, pax−/− cells extended few lateral lamellipodia at early time points, and their CDRs formed more slowly and persisted for longer times before being internalized (Fig. 8B). The internalized vesicles also persisted beyond 30 min and typically remained at the cell periphery instead of translocating rapidly to the perinuclear region.

These results were confirmed in cells fixed at various times after stimulation. About 40% of pax+ cells had a single CDR and 15% had two or more at 5 min (Fig. 8C, D), whereas more than 90% of PDGF-stimulated pax−/− cells formed CDRs (Fig. 8C), and about 40% had two or more per cell (Fig. 8D). At 30 min, about 17% of pax−/− cells were positive for CDRs (Fig. 8E), and time-lapse analysis revealed that pax−/− cells underwent successive rounds of CDR formation over 45 min (Fig. 8B). Similar excessive CDR formation was also observed in paxN cells (Fig. 8E) with over 80% of paxN cells exhibiting CDRs at 5 min after PDGF addition (Fig. 8C). Consistent with the larger area of dorsal protrusions observed in cells on square ECM islands, more than half of these cells formed two or more CDRs (data not shown). In contrast, only about 30% of paxC cells, had CDRs at 5 min (Fig. 8C), which was significantly less than pax+ cells (*p*<0.01), and none had CDRs at 15 or 30 min (data not shown).

### Paxillin is required for efficient directional migration in 2D

**Figure 9:**
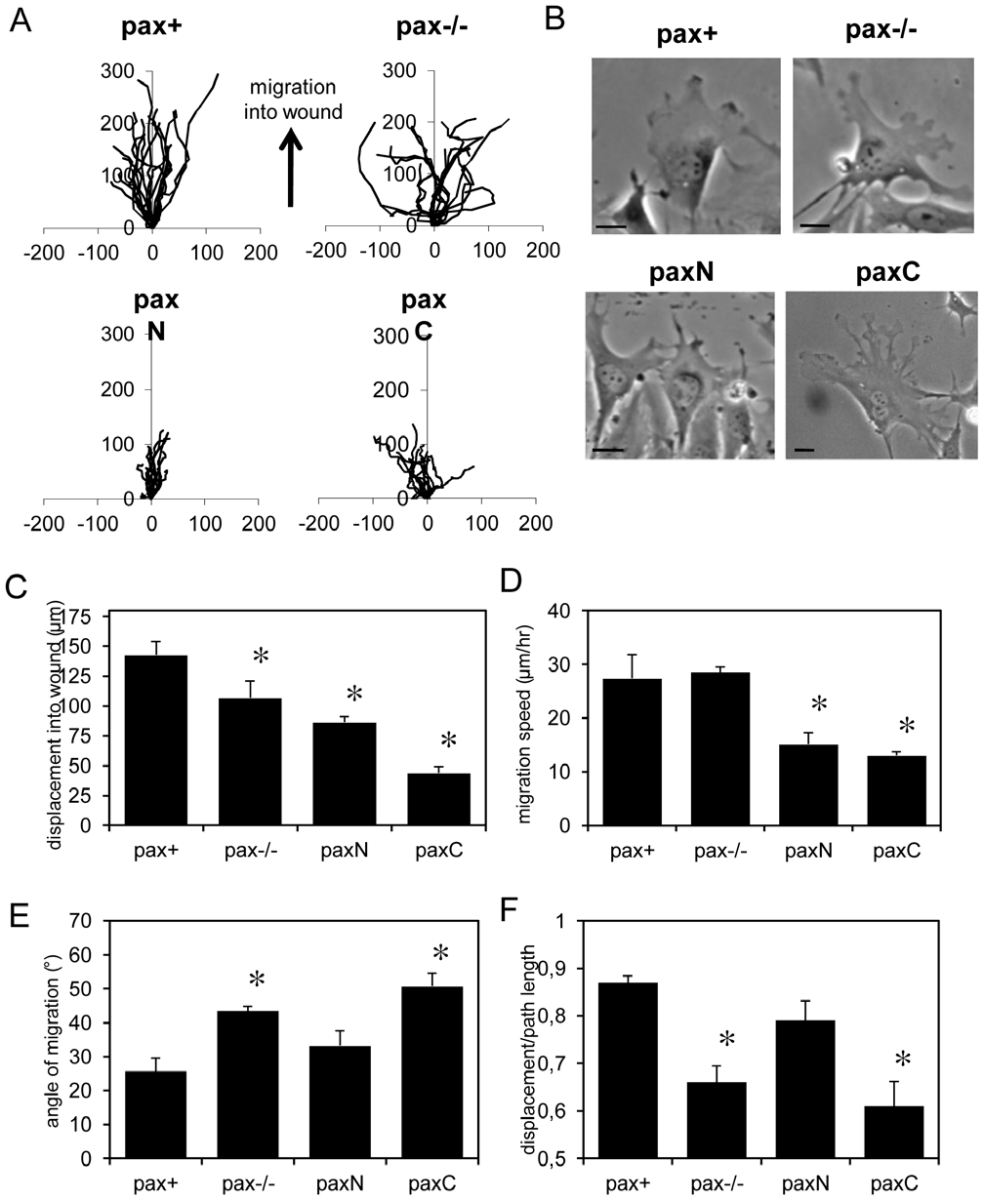
Paxillin deficiency leads to loss of directional persistence, but not migration speed; paxN rescues directional migration into scrape wounds, but expression of either half of paxillin alone results in decreased cell speed. A) Paths of individual cells at the edge of scrape-wounds over 6 h. B) Representative images of cells at the wound edge. Note thin, spiky protrusions in the paxN cell and multiple lamellipodia in pax−/− and paxC cells. Scale bar = 10 µm. C) Displacement into the scrape wound after 6 h. D) Migration speed of cells migrating into scrape wounds. E) Average angle of migration into scrape wounds (90° = perpendicular to wound edge). F) Displacement/path length ratio (1 = perfectly straight path into wound). * *p*<0.001 compared to pax+.

Paxillin loss and mutation have been reported to induce migration defects, including impaired movement in a 2D scrape-wound assay [36]. Computerized tracking of cell migration paths over a period of 6 hours after scrape wounding confluent cell monolayers confirmed that pax−/− cells migrated in a less coordinated fashion (Fig. 9A, top) and that the average displacement into the wound was significantly lower for pax−/− cells than control pax+ cells (*p*<0.01) (Fig. 9C). Importantly, this was not due to reduced migration speed (Fig. 9D), but to impairments in the average angle of migration (Fig. 9E) and directional persistence (displacement/path length ratio) (Fig. 9F). Similar results were obtained for control and pax k/d HDFs (Fig. S5). Thus, the defect in spatial regulation of lamellipodia extension seen in paxillin-deficient cells on square ECM islands correlates with a defect in directional migration in response to another physical cue associated with loss of contact inhibition.

From the results of the square cell assays, we expected that paxN would rescue directional migration into the scrape wound, whereas paxC would not. Single-cell tracking confirmed this hypothesis (Fig. 9A, bottom), but also revealed that expression of either truncation mutant alone resulted in reduced migration speed (Fig. 9D). The average displacement of paxN cells into the wound was significantly lower than that of pax+ cells (*p*<0.0005), but similar to pax−/− cells (Fig. 9C). Although the migration speed of paxN cells was significantly reduced (*p*<0.0001) (Fig. 9D), their average angle of migration was similar to controls (Fig. 9E) and they maintained directional persistence (Fig. 9A, F). The average displacement into the wound of paxC cells was much lower than that of both pax+ (*p*<0.00001) and pax−/− (*p*<0.0001) cells, and about half that of paxN cells (Fig. 9C). This was due to both reduced migration speed (*p*<0.0001) (Fig. 9D) and loss of directional persistence (Fig. 9A,E,F). Similar differences in directional persistence were observed for randomly migrating cells as well (data not shown).

The morphology of migrating cells was consistent with what we observed in cells on square islands. Pax−/− cells at the wound edge initially polarized toward the open wound but then formed multiple leading edges oriented in different directions as they migrated (Fig. 9B). Furthermore, many cells also formed CDRs in the first two hours after wounding, and several of these formed multiple CDRs. PaxN cells remained oriented toward the wound edge, but extended spiky, phase-dark protrusive structures rather than broad leading edges, and a few paxN cells at the wound edge also formed CDRs. PaxC cells tended to be larger with multiple leading edges. Pax+ cells also formed some spiky, phase-dark protrusions and a few formed CDRs (3 out of 49 cells at the wound edge), but they tended to maintain leading edges facing the wound space and polarized morphologies over several hours.

### Paxillin regulates cell invasion through ECM in 3D

While the effects of paxillin mutation on cell migration have been studied in 2D, little is known about its role in invasion through 3D ECMs, which is likely more physiologically relevant for development, wound healing, and cancer metastasis. The finding that pax−/− and paxN cells have an increased propensity to form CDRs was particularly intriguing in this regard, as it has been proposed that CDRs are related to invasive protrusions [52], and paxillin has been implicated in cancer progression [29], [30]. We therefore carried out 3D migration studies using a modified Transwell invasion assay in which cells were plated on the lower surfaces of inserts that were filled with ∼100 µm thick Matrigel plugs [53], and then stimulated (or not) by adding PDGF to the upper chamber.

**Figure 10:**
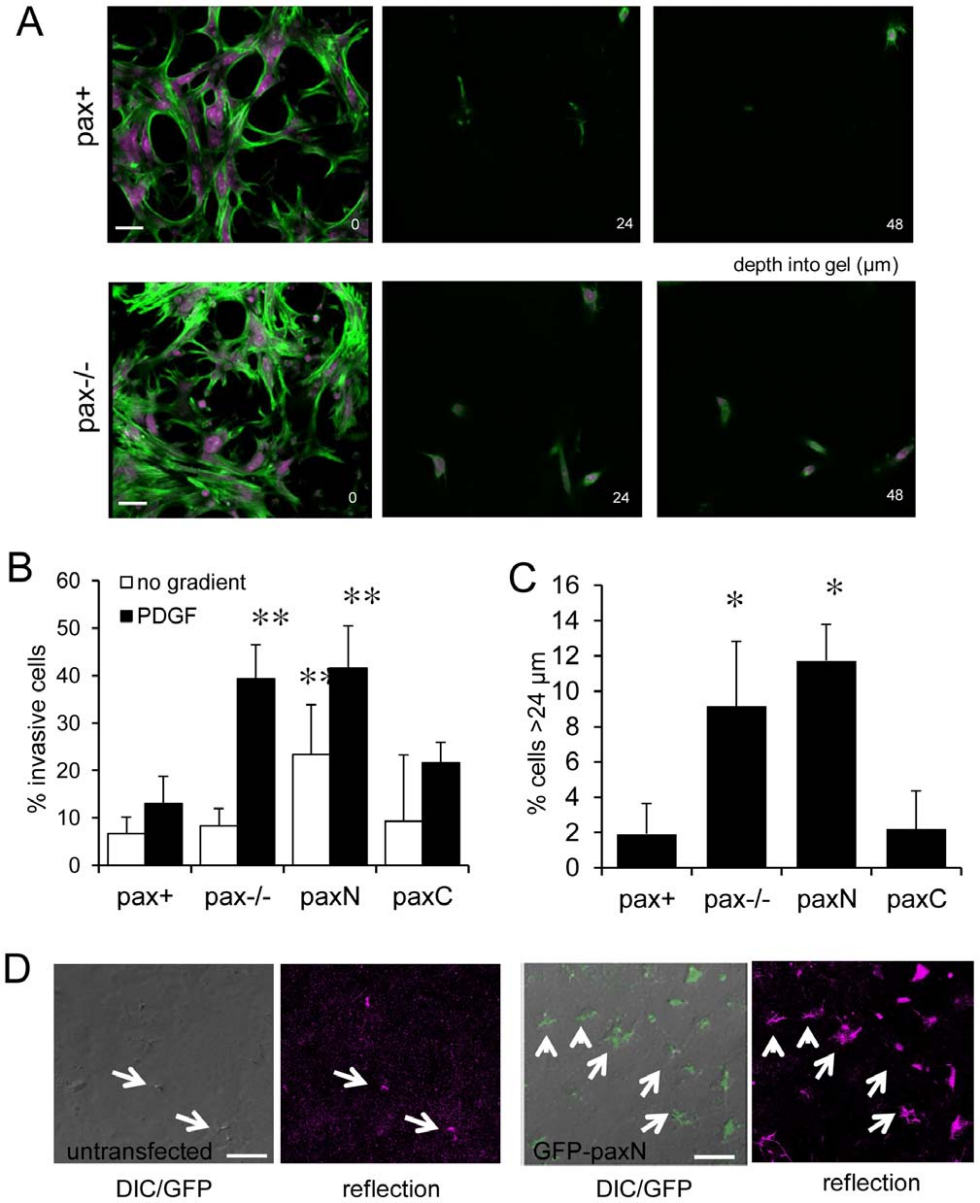
Loss of paxillin leads to increased migration into 3D Matrigel plugs, and paxN and paxC have opposite effects on matrix invasion. A) Representative images of cells at various depths (µm) into Matrigel plugs, stained for actin (green) and DNA (magenta). Cells at 0 µm are on the upper surface of Transwell inserts, just below the 3D matrix. Scale bar = 10 µm. B) Percent of cells with nuclei at heights greater than 8 µm into Matrigel plugs, with or without PDGF gradients (25 ng/ml PDGF added to the top of the insert well). n>1000 cells per genotype. ** *p*<0.001 compared to pax+. C) Percent of cells with nuclei at depths greater than 24 µm into Matrigel plugs in the presence of PDGF gradients. * *p*<0.05 compared to pax+. D) Expression of GFP-paxN drives invasive protrusion formation in response to PDGF in pax+ cells. DIC, fluorescence (green), and confocal reflection imaging (magenta) show protrusions (white arrows) in cells overlaid with Matrigel stimulated with PDGF (25 ng/ml) for 2 hours. Scale bar = 10 µm.

MEFs cultured for 24 hours on the lower surfaces of Transwell inserts were able to migrate across the porous membranes, as indicated by the appearance of flat, spread cells below the Matrigel (Fig. 10A, left). From confocal sections (4 µm spacing, 0–60 µm from the Transwell surface) and reconstructed images of confocal z-stacks (Fig. S6), cells were scored as “invasive” if their nuclei were located more than 8 µm deep in the Matrigel plugs. Pax+ and paxC cells were only minimally invasive, even in the presence of a PDGF gradient (Fig. 10A, top, and 10B), whereas pax−/− cells invaded the ECM to a 3-fold greater degree than controls when stimulated with PDGF (Fig. 10A, bottom, and 10B; *p*<0.001). PaxN cells were also significantly more invasive than control (pax+) cells when stimulated with PDGF (*p*<0.001) (Fig. 10B). Furthermore, both pax−/− and paxN cells stimulated with PDGF invaded the Matrigel to greater depths than did pax+ or paxC cells (Fig. 10A, C), with more than four times as many pax−/− and paxN cells being observed at depths greater than 24 µm (roughly the half-way point of the z-stacks) compared to pax+ cells (Fig. 10C; *p*<0.05). Actin-rich processes extended by pax−/− and paxN cells were even detected as far as 60 µm or more into the gels. Most interestingly, paxN cells invaded the Matrigel plugs even in the absence of a growth factor gradient (Fig. 10B).

To test whether the paxN fragment could actively promote ECM invasion, confluent monolayers of pax+ cells with or without GFP-paxN were overlaid with Matrigel and stimulated with PDGF. Spiky protrusions extended up into the Matrigel from the surfaces of GFP-paxN cells after 2 hrs, as shown by differential interference (DIC) and confocal reflection imaging (Fig. 10D). Consistent with the results of the invasion assay, cells expressing paxN formed more extensive projections into the overlying Matrigel than untransfected cells (Fig. 10D). These protrusions likely correspond to the CDRs seen on the apical surfaces of cells on 2D substrates.

## Discussion

Culturing cells on microfabricated ECM substrates allowed us to control cytoskeletal polarity and FA position, which enabled us to predict where lamellipodia were likely to form when cells were stimulated by a soluble motility factor. Using this system, we were able to detect uncoupling of membrane extension from spatial cues and to analyze the role of paxillin subdomains in this motile response. Normal mouse and human fibroblasts plated on single cell-sized, square FN islands formed large FAs primarily in corner regions and preferentially extended lamellipodia from adjacent sites in response to PDGF. In contrast, paxillin-deficient cells formed more and smaller FAs as well as lamellipodia along the cell periphery, with little spatial preference. These results indicate that paxillin is involved in both promoting membrane extension near FAs, as well as suppressing lamellipodia formation at distant sites.

In addition to showing that paxillin is critical for spatially coupling regions of cell distortion and sites of FA assembly to sites where new lamellipodia will form, we found that the N- and C-termini of paxillin play opposing, but complementary, roles in this process (Table 1). The N-terminus is critical for suppressing lamellipodia formation and maintaining directional persistence, while the C-terminus actively promotes lamellipodia formation. An unexpected finding was that paxillin mutation also affects the formation of dorsal CDRs, as well as lateral membrane extensions. Most importantly, these studies revealed that in addition to regulating directional migration in 2D, paxillin is a critical mediator of ECM invasion and migration in 3D, and this more complex response correlates with formation of CDRs in 2D cultures.

**Table 1:** Phenotypes of paxillin mutants (MEFs).

	CDR (5 min)	Corner lamellipodia (30 min)	Side lamellipodia (30 min)	Direction persistence (2D)	Matrigel invasion (3D)
pax+	++	+++	+	+++	+
pax−/−	+++	++	++	+	++
paxN	+++	++	+	+++	+++
paxC	+	++++	++	+	+

Cells migrating on ECM substrates that vary in the mechanical compliance (flexibility) move in the direction in which they exert the highest traction forces [4]. In square-shaped cells, traction forces are concentrated in corner regions [9], likely due to positive feedback between geometric constraints and contractility-dependent assembly of FAs [12], [54], [55], [56], [57]. In addition to containing high concentrations of signaling molecules, FAs may be “permissive zones” for membrane extension in that actin-driven protrusions are not blocked by cortical actin (due to stress fiber insertions). In support of this hypothesis, myosin-mediated cortical tension has been shown to inhibit branching in endothelial cells, and inhibition of myosin II subjacent to the plasma membrane can induce localized membrane protrusion [58].

At early time-points after PDGF stimulation (5–10 min), cells with and without paxillin formed both dorsal and lateral membrane extensions. This suggests that paxillin is not required for the initial burst of actin-driven ruffling in response to growth factor stimulation, and that this early process may be molecularly distinct from later rounds of lamellipodia formation. We also found that pax −/−cells and cells expressing the paxN truncation mutant had a greater propensity to form CDRs on their apical membranes than pax+ or paxC cells. CDRs frequently form over the leading edges of motile cells and contain many of the same protein components as lamellipodia (e.g., actin, Arp2/3, vinculin, paxillin; Fig. S2B, C and D); however, they have been shown to be structurally and biochemically distinct protrusive structures [59], which is consistent with our findings.

Although many paxillin domains have been studied, little is known about the conformation of paxillin *in vivo*. Vinculin in FAs undergoes a conformational change that relieves an intramolecular association between the head and tail regions, exposing protein-protein interaction domains that are hidden in the cytosolic form [60]. Like vinculin, paxillin may adopt different conformations upon FA recruitment that expose or sequester various protein-interaction sites, which could explain the complex effects of the truncation mutants on the formation of different protrusive structures. It is possible that the different effects of the paxN and paxC truncation mutants are due to exposure of binding domains that are usually only available in specific subcellular contexts (e.g. in FAs versus the cytosol). The paxN and paxC truncation mutants may thus act as “dominant negatives”, sequestering proteins away from other binding partners, or “dominant positives” that can interact with proteins that normally would be unavailable in a given subcellular context.

Paxillin binds the ArfGAPs Git1 and Pkl/Git2 via its N-terminal LD4 motif [61], and these proteins have been implicated in directional motility through both positive and negative mechanisms. Git-1 has been reported to either inhibit membrane extension [62], [63] or promote cell migration depending on its location within the cell [64], whereas Pkl appears to be involved in control of directional cell migration in fibroblasts [65], [66]. Localization of Pkl to FAs is regulated by tyrosine phosphorylation, and its dephosphorylation is mediated by PTP-PEST, which binds to paxillin via its C-terminal LIM domains [49]. Thus, the two halves of paxillin may work together to efficiently drive directional migration by mediating complex cycles of these types of molecular associations.

These findings indicate that paxillin may play distinct roles in different subcellular contexts, such as regulating the formation of different kinds of motile processes (e.g., broad or fan-shaped lamellipodia, filopodia, lateral versus dorsal ruffles). These data also suggest cytoplasmic functions for paxillin in controlling CDR extension and membrane trafficking, as well as lamellipodia formation, which may correspond to different modes of migration *in vivo* [67]. Interestingly, CDRs formed by mesenchymal cells in 2D have been compared to invasive protrusions or ‘invadopodia’ formed by epithelial cells [42], [52], [59]. Cells in tissue culture have basal membranes that are in contact with ECM and free dorsal surfaces, whereas mesenchymal cells are usually embedded within 3D ECMs in tissues. We found that dorsal protrusion formation in MEFs correlated with the ability of these cells to invade 3D Matrigel plugs. Paxillin-mediated signaling may therefore be critical for determining whether a cell migrates along a planar (2D) basement membrane or through a 3D interstitial matrix, as occurs, for example, during epithelial-mesenchymal transitions in cancer metastasis. Moreover, switching between Rho- and Rac-mediated modes of migration (e.g. ameboid versus mesenchymal) is a common feature of 3D matrix invasion [53], [67], [68], [69]. Thus, paxillin may be involved in tailoring a cell's motile response to physical cues in different microenvironments. Importantly, mutation and misregulation of paxillin correlate with metastatic potential in some human breast and lung cancers [28], [30], suggesting that it may be involved in regulating ECM invasion as well. In any case, the different effects of paxillin deficiency in 2D versus 3D migration underscore the importance of the physical microenvironment on cell behavior, and the central role that paxillin normally plays in this process.

In conclusion, detailed examination of fibroblast cells on patterned and unpatterned substrates revealed that they respond to PDGF with an initial round of membrane extension in all directions, followed by progressive spatial fine-tuning that is sensitive to physical cues. Thus, lamellipodia formation becomes preferentially localized to regions of greatest cell distortion (i.e., corners) after 15 min of PDGF stimulation in these artificially polarized cells. We found that paxillin can enhance or suppress membrane extension depending on its subcellular context. The presence of paxillin within FAs appears to spatially constrain where Rac is activated inside the cell, and thereby preferentially stimulates motile process formation to adjacent regions. Loss of paxillin results in deregulated spatial pruning of membrane extensions. The N-terminus appears to suppress lateral membrane extension, and the C-terminus enhances lamellipodia formation, but both halves are required for efficient directional migration in 2D. Furthermore, overexpression of the N- or C-terminus alone can tip the balance between “dorsal” and “lateral” motile process formation in response to PDGF. Taken together, these results shed light on the molecular mechanism by which cell motility is directed by its physical microenvironment, in addition to revealing new functions for paxillin in coordinating cell migration in both 2D and 3D that might be highly relevant for developmental control *in vivo*.

## Materials and Methods

### Cell culture and media

Primary MEFs derived from E7.5 embryos [36], NIH 3T3 cells, and primary HDFs (isolated from human neonatal foreskin) were grown in high glucose DMEM (Gibco) supplemented with 15% FBS (MEFs) or 10% FBS (3T3 and HDFs) (HyClone) and 1% penicillin/streptomycin/L-glutamine (P/S/G) (Gibco). IMR-90 cells (ATCC) were grown in MEM (ATCC) supplemented with 10% FBS (ATCC) and 1% P/S/G. Serum-free defined medium (DM) consisted of basal medium (DMEM or MEM) plus 1% P/S/G and 1% BSA (Chemicon). Paxillin −/− MEFs were infected with retrovirus containing myc-paxillin, myc-paxN, or myc-paxC and selected in puromycin [36]. We used myc-tagged paxillin to rescue pax−/− cells as controls instead of wild-type MEFs because commercial antibodies to paxillin also recognize the FA protein Hic-5.

Recombinant human PDGF-BB was obtained from BioVision. Fibronectin (BD Biosciences) was freshly prepared from 5 mg/mL aqueous stock solution (stored at −80°C) before stamping. Tissue culture plates were coated with 0.6 ng/cm^2^ in carbonate/bicarbonate buffer (0.1 M Na_2_HCO_3_, 0.1 M NaH_2_CO_3_, pH 9.4) for wound heal experiments. For live cell imaging experiments, cells were plated in microscopy medium: H-MEM (Phenol Red-free MEM plus HEPES) supplemented with 1× MEM vitamins, 1% P/S/G, and either 1% BSA or FBS.

### Microcontact printing

Stamps were created using soft lithography as described previously [70], [71]. PDMS stamps were made by casting the polymer onto silicon wafers that have been etched by photolithography with corresponding microscale features. Substrates for stamping were fabricated by spin-coating a thin layer of PDMS (Sylgard-184, Dow Corning) onto glass coverslips. To coat a coverslip or coverslip-bottomed petri dish (MatTek), a drop of PDMS (200 µl for a 25 mm×25 mm coverslip, Corning) was applied to the center of the coverslip and spun at 4000 rpm for 4 minutes on a spin-coater (Specialty Coating Systems G3P-8, Cookson Electronics) and cured at 60°C for one hour.

Prior to stamping, PDMS stamps were cleaned in 70% ethanol in a sonicating water bath for 30 minutes, rinsed with water, and dried using filtered compressed air or nitrogen gas. The surface of the clean stamps containing the raised micropatterned features were incubated with 50 µg/mL FN in aqueous solution for one hour, and dried thoroughly with filtered nitrogen gas or compressed air. Directly before use, the PDMS-coated coverslips were activated by oxygen plasma in a UVO cleaner (Jelight) for 8 minutes, during which time inked PDMS stamps were dried. The stamps were then pressed gently against the plasma-treated PDMS surface to ensure complete contact of stamp with substrate. Unstamped areas were blocked by incubation in an aqueous solution of 1% Pluronic-127 for 1 hour at room temperature or overnight at 4°C. Before plating cells, substrates were washed three times with PBS to remove residual Pluronic.

### Quantification of membrane extension and FAs in square cells

Cells on square-stamped substrates were fixed and stained for actin using Alexa 488-phalloidin and FN and vinculin by immunostaining. Membrane extensions were defined as actin-rich structures emanating from the cell periphery with areas greater than 1 µm^2^. Images of single cells on stamped islands were overlaid with a 4×4 grid to divide the cell into corner and side regions of equal perimeter (Fig. 1B). Images were subjected to intensity thresholding, bright regions of membrane extension were outlined to define regions of interest (ROIs), and the projected area of each ROI was measured using IPLab (Scanalytics) image analysis software. Corner extensions were defined as those that emanated from the cell perimeter in corner regions, and side extensions as any extensions originating from side regions. The total distance of corner- and side-defined regions along the cell periphery were equal. FAs in regions defined as corner or side, with equal area and edge lengths (Fig. 8A), were counted and measured using IPLab.

### Antibodies and reagents

The following primary antibodies were used for immunostaining and Western blot analysis: anti-paxillin mouse monoclonal (BD Biosciences), anti-vinculin mouse monoclonal (BD Biosciences), anti-fibronectin rabbit polyclonal (Sigma), anti-myc (9E10) mouse monoclonal (Upstate), anti-GAPDH mouse monoclonal (Chemicon). Goat anti-mouse and anti-rabbit Alexa-488 and Alexa-594 conjugated secondary antibodies, DAPI, and Alexa-488 and Alexa-594 phalloidin were obtained from Molecular Probes (Invitrogen).

### Immunostaining

Cells on micropatterned substrates were initially fixed in 4% paraformaldehyde (PFA) in PBS for 15 min at room temperature (RT). However, the integrity of delicate protrusive structures was better preserved by using a fixation buffer containing 100 mM PIPES (pH 6.8), 1 mM EGTA, 1 mM MgSO_4_, 2 mM glycerol, and 4% PFA. This buffer was warmed to 37°C before use. Cells were incubated in warmed buffer for 15 min at RT and washed 3× with PBS. Cells were permeabilized and blocked in IF buffer (0.1% Triton X-100, 0.5% BSA in PBS) for 10 min before staining. Primary and secondary antibodies were diluted in IF buffer and coverslips were inverted on 50–100 µl drops of antibody solution on Parafilm and incubated at RT for 1 h. Washes were performed with IF buffer, as the presence of detergent helped prevent desiccation. DAPI (Molecular Probes) was added for 5 min before the last wash. Coverslips were mounted on slides using Fluoromount-G (Southern Biotechnology).

### DNA and RNA constructs and transient transfection

GFP-paxillin (chicken paxillin in Clonetics pEGFP-C vector) was transiently transfected into 3T3 cells using Effectene (Qiagen) according to the manufacturer's protocol. MEFs were transfected using the Amaxa nucleofection system using MEF Kit 2 buffer (Amaxa) on setting T-20.

GFP-paxillin (Clontech EGFP-C, chicken paxillin mRNA) constructs were grown in XL1-Blue *E. coli* (Stratagene) under kanamycin selection for all cloning steps. To generate GFP-paxN, GFP-paxillin (∼6.4 kb) was digested with *BbsI* and *EcoRI* and the ∼5.6 kb fragment, containing GFP and paxillin residues 1–345, was ligated using a linker oligonucleotide (gg cac *ctcgag* tag ggc) (IDT) engineered with a STOP codon (underlined) and a novel *XhoI* site (italics) to facilitate identification of the insert. GFP-paxC was constructed by digesting the GFP-paxillin construct with *BspEI* and *BbsI*. The resulting ∼5.4 kb fragment was ligated to a 111 bp oligonucleotide linker, digested from a pZero 2 cloning vector (IDT) using the same enzymes, to generate a ∼5.5 kb construct composed of the GFP tag and residues 308–599 of paxillin. Restriction enzymes were obtained from New England Biolabs.

The Raichu-Rac1 FRET construct was kindly provided by Dr. Michiyuki Matsuda (Osaka University, Osaka, Japan).

siRNA against human paxillin, (gtg tgg agc ctt ctt tgg t) [37] was obtained as a custom oligonucleotide dimer from Ambion.

### Microscopy and time-lapse imaging

Real-time recording of cells was carried out using a Hamamatsu CCD camera on a Nikon Diaphot 300 inverted microscope equipped with phase contrast optics and epifluorescence illumination, and processed using the computerized image acquisition and analysis tools of IPLab Spectrum (Scanalytics, Fairfax, VA). The microscope was equipped with an on-stage heater that maintained the temperature at 37°C. To prevent evaporation of water, the culture medium was covered with a thin layer of mineral oil. High-resolution immunofluorescence images were also acquired using a Leica TCS SP2 confocal laser scanning microscope.

### Western blotting

Cells were lysed in 1% Triton X-100 lysis buffer (25 mM Tris pH 7.4, 150 mM NaCl, 1 mM EDTA, 1% Triton X-100) plus protease inhibitor and phosphatase inhibitor cocktails (Pierce). Lysates were run on NuPAGE Bis-Tris gels in 1× Laemmi's SDS buffer using a Novex gel electrophoresis system and transferred to nitrocellulose membranes using NuPAGE transfer buffer with 1–10% methanol for 2–3 h at 22V. Blots were blocked with TBST with 5% non-fat dry milk for 1 h at RT and probed with primary antibody for 1 h at RT or overnight at 4°C. After washing in TBST, blots were incubated for 1 h at RT with HRP-conjugated secondary antibodies (Vector Laboratories) and washed. Bands were visualized using SuperSignal Dura West ECL reagent (Pierce).

### Matrigel invasion assay

Matrigel invasion assays were performed following the protocol described by Sahai and Marshall [53]. First, 50 µl of growth factor-reduced, phenol red-free Matrigel (BD Biosciences) was plated into top chambers of Transwell inserts (8-µm pore size, 6.5 mm diameter; Costar) and allowed to gel at 37°C for 30 min. Cells were tryspinized, counted, and resuspended in DMEM plus 5% FBS and 5 µg/ml FN at a concentration of 1×10^6^ cells/ml. Transwell inserts containing Matrigel plugs were then inverted and 50 µl of cell suspension was added to the bottom of each and incubated for 2 h to allow cells to adhere. Inserts were placed into 24-well plates containing 300 µl DMEM plus 5% FBS in the lower chamber. 200 µl of the same medium was added to the top of each insert containing the Matrigel plug. After 24 h, PDGF in DMEM (25 ng/ml final concentration) or DMEM alone was added to the top chambers and incubated for a further 16 h.

Inserts were washed twice with PBS and fixed in PBS plus 4% paraformaldehyde for 30 min at RT. Inserts were then washed 3× with PBS, with 10 min incubations (RT) for each wash, and permeabilized with Matrigel IF buffer (130 mM NaCl, 7 mM Na_2_HPO_4_, 3.5 mM NaH_2_PO_4_, 7.7 mM NaN_3_, 0.1% BSA, 0.2% Triton X-100, 0.05% Tween-20) for 30 min (RT). Actin and nuclei were visualized by staining with 1∶200 dilutions of Alexa594-phalloidin (200 U/ml; Molecular Probes) and DAPI (5 mg/ml; Molecular Probes) in Matrigel IF buffer at 37°C for 1 h. Prior to imaging, inserts were washed 2× with ddH_2_O.

Imaging was performed on a Leica TCS SP2 laser scanning confocal microscope by situating the Transwell insert in a coverglass-bottom dish (MatTek) and acquiring z-stacks from the bottom of the porous insert up through the Matrigel plug at 4 µm intervals (using 2× line average and 3× frame average settings to reduce background). At least five fields of view (FOV) were counted for each insert, and all conditions were done in duplicate or triplicate. DAPI staining was best visualized using the 63×/1.4 NA oil objective lens.

Quantification was performed by counting the number of nuclei in each z-stack for each FOV. Cells were scored as “invasive” if nuclei were more than 8 µm into the Matrigel plug and expressed as percentage of total cells that were invasive. 500–1000 total cells were counted for each condition in each experiment, and experiments were repeated in duplicate (paxN) or in triplicate (pax+ and pax−/−).

### Confocal reflection microscopy

Confocal reflection microscopy was performed following the method of Friedl and colleagues [72], [73], [74] in conjunction with DIC and fluorescence microscopy to detect invasive cellular protrusions and Matrigel remodeling. Gels were illuminated with 594 nm laser light and the emission gate was set to 590–600 nm. Offset in the red channel was set to −16 to enhance contrast. DIC and GFP fluorescence were imaged simultaneously using standard fluorescence and DIC settings.

## Supporting Information

Figure S1 **Microcontact printing method.** A) A PDMS stamp cast from a photolithographed silicon master wafer is inked with an aqueous solution of protein, e.g. fibronectin (FN), and dried using compressed air or N_2_. B) A glass cover-slip spin-coated with a thin layer and PDMS is treated by plasma oxidation to activate the surface. C) The inked stamp is brought into conformal contact with the oxidized substrate for 1 min and removed, transferring the protein from the raised features of the stamp to the activated PDMS surface. Unstamped areas are made non-adhesive by incubating in a 1% solution of Pluronic F-127. D) The stamped substrate is washed with PBS and cells are plated. Cells adhere only to microcontact-printed adhesive islands.
(TIF)

Figure S2 **Circular dorsal ruffles are induced by PDGF stimulation and contain paxillin.** A) paxN (left) and C (right) MEFs plated on 50×50 µm FN islands, fixed at 5 min after stimulation with PDGF, and stained with Alexa488-phlloidin to label F-actin. B) GFP-paxillin localizes to CDRs at 5 min after PDGF stimulation. C and D) Human dermal fibroblasts stimulated with PDGF for 5 min (C) or 30 min (D), stained with Alexa488-phalloidin and p34 (Arp2/3).
(TIF)

Figure S3 **Focal adhesion size and distribution in paxN and paxC cells.** A) PaxN (top) and paxC (bottom) cells labeled with anti-myc and anti-vinculin antibodies. B) Number of FAs per region. C) Average lengths of FAs in each region. * *p*<0.01 compared to pax+.
(TIF)

Figure S4 **Expression of GFP-paxillin, GFP-paxN, and GFP-paxC in pax−/− cells.** A) Localization of transiently transfected GFP-tagged constructs expressed in pax−/− MEFs. Scale bar = 10 µm. B) Average extension areas in corners and sides of pax−/− cells expressing GFP or GFP-tagged paxillin constructs. * *p*<0.01 compared to GFP. C) Western blot of GFP-paxillin (pax), GFP-paxN, and GFP-paxC expressed by transient transfection in pax−/− MEFs. The mutant genes were cloned from the same construct carrying the full-length gene (see Materials and Methods), equal amounts of plasmid were used to transfect three aliquots of the same pax−/− cells, and equal amounts of total protein (∼30 µg) were loaded.
(TIF)

Figure S5 **Knockdown of paxillin in human dermal fibroblasts leads to loss of directional persistence but not migration speed.** A) Paths of individual cells at scrape wound edges over 6 h. B) Displacement into wounds. C) Migration speed. D) Average angle of migration. E) Displacement/path length, i.e. directional persistence. * *p*<0.001 compared to control.
(TIF)

Figure S6 **3D reconstruction of invasive pax−/− cells in Matrigel.** A) side-view of stack. B) Top-view of stack. Actin is labeled in green, DNA in blue. Length of grid unit = 12 µm.
(TIF)
