# Supplementary figures and images for: Paxillin Mediates Sensing of Physical Cues and Regulates Directional Cell Motility by Controlling Lamellipodia Positioning
Source: PLoS One. 2011 Dec 14;6(12):e28303. doi: 10.1371/journal.pone.0028303 (PMC3237434; doi:10.1371/journal.pone.0028303)

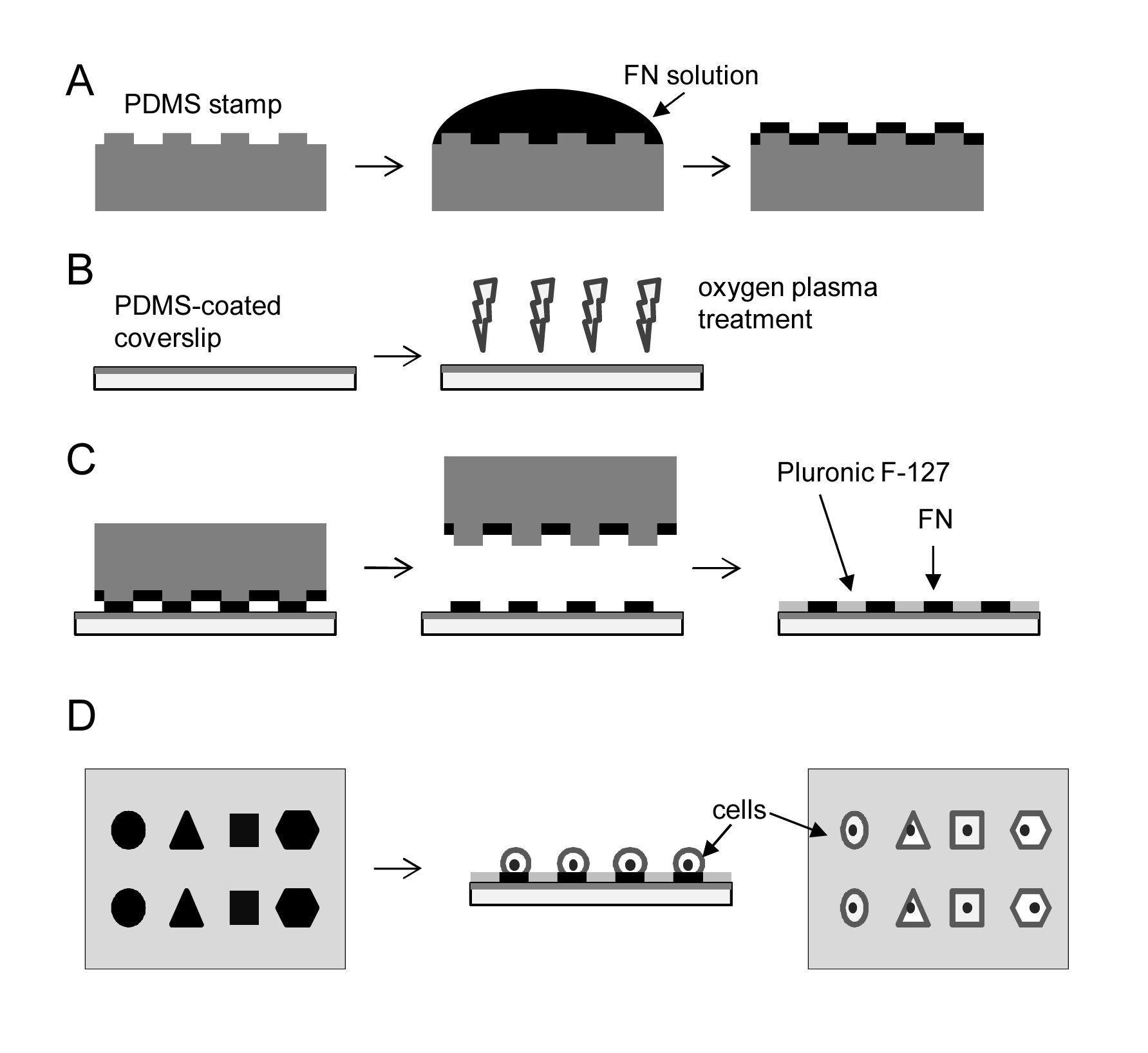

Supplement: Figure S1 — Microcontact printing method. A) A PDMS stamp cast from a photolithographed silicon master wafer is inked with an aqueous solution of protein, e.g. fibronectin (FN), and dried using compressed air or N2. B) A glass cover-slip spin-coated with a thin layer and PDMS is treated by plasma oxidation to activate the surface. C) The inked stamp is brought into conformal contact with the oxidized substrate for 1 min and removed, transferring the protein from the raised features of the stamp to the activated PDMS surface. Unstamped areas are made non-adhesive by incubating in a 1% solution of Pluronic F-127. D) The stamped substrate is washed with PBS and cells are plated. Cells adhere only to microcontact-printed adhesive islands. (TIF) [file pone.0028303.s001.tif]

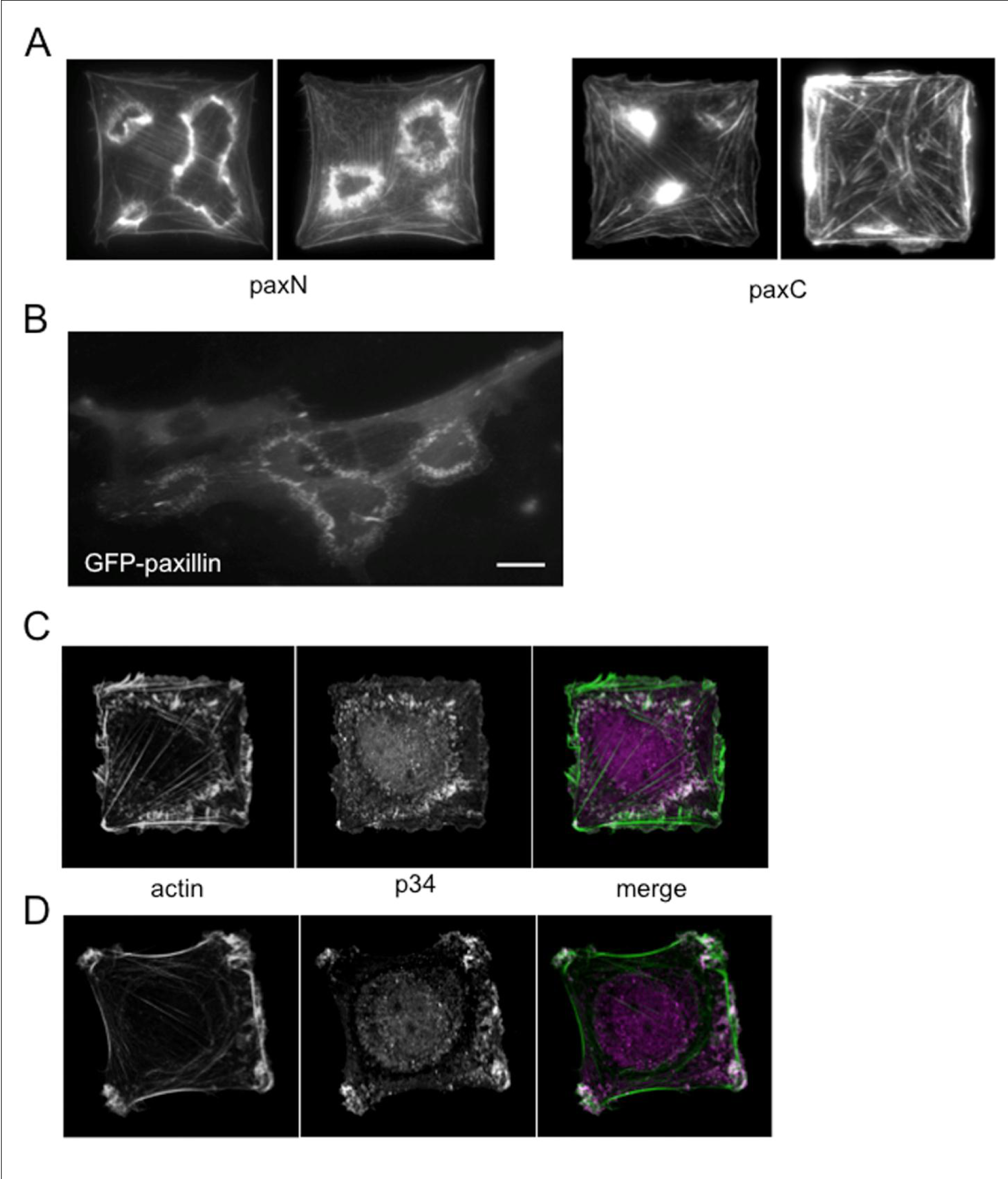

Supplement: Figure S2 — Circular dorsal ruffles are induced by PDGF stimulation and contain paxillin. A) paxN (left) and C (right) MEFs plated on 50×50 µm FN islands, fixed at 5 min after stimulation with PDGF, and stained with Alexa488-phlloidin to label F-actin. B) GFP-paxillin localizes to CDRs at 5 min after PDGF stimulation. C and D) Human dermal fibroblasts stimulated with PDGF for 5 min (C) or 30 min (D), stained with Alexa488-phalloidin and p34 (Arp2/3). (TIF) [file pone.0028303.s002.tif]

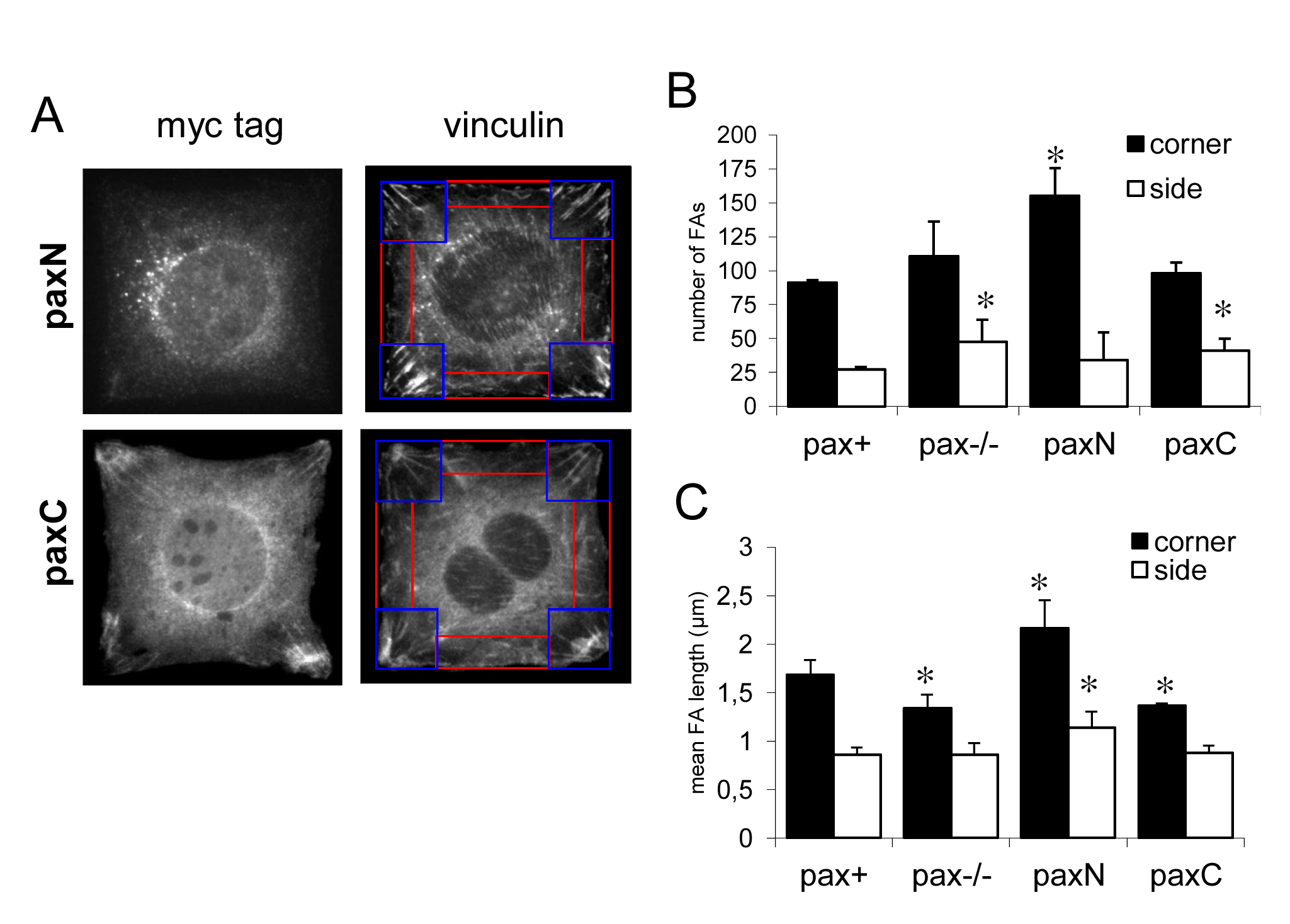

Supplement: Figure S3 — Focal adhesion size and distribution in paxN and paxC cells. A) PaxN (top) and paxC (bottom) cells labeled with anti-myc and anti-vinculin antibodies. B) Number of FAs per region. C) Average lengths of FAs in each region. * p<0.01 compared to pax+. (TIF) [file pone.0028303.s003.tif]

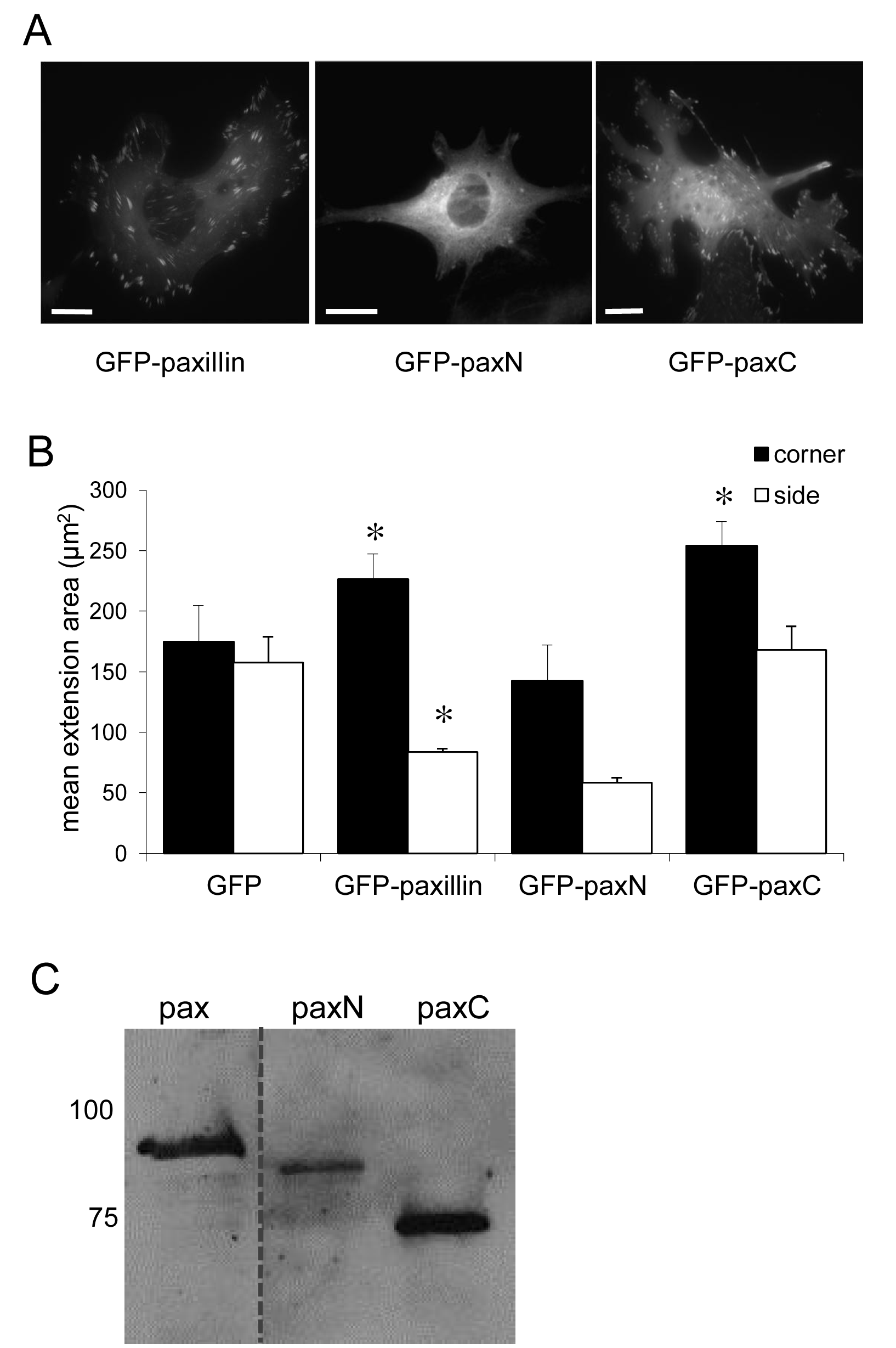

Supplement: Figure S4 — Expression of GFP-paxillin, GFP-paxN, and GFP-paxC in pax−/− cells. A) Localization of transiently transfected GFP-tagged constructs expressed in pax−/− MEFs. Scale bar = 10 µm. B) Average extension areas in corners and sides of pax−/− cells expressing GFP or GFP-tagged paxillin constructs. * p<0.01 compared to GFP. C) Western blot of GFP-paxillin (pax), GFP-paxN, and GFP-paxC expressed by transient transfection in pax−/− MEFs. The mutant genes were cloned from the same construct carrying the full-length gene (see Materials and Methods), equal amounts of plasmid were used to transfect three aliquots of the same pax−/− cells, and equal amounts of total protein (∼30 µg) were loaded. (TIF) [file pone.0028303.s004.tif]

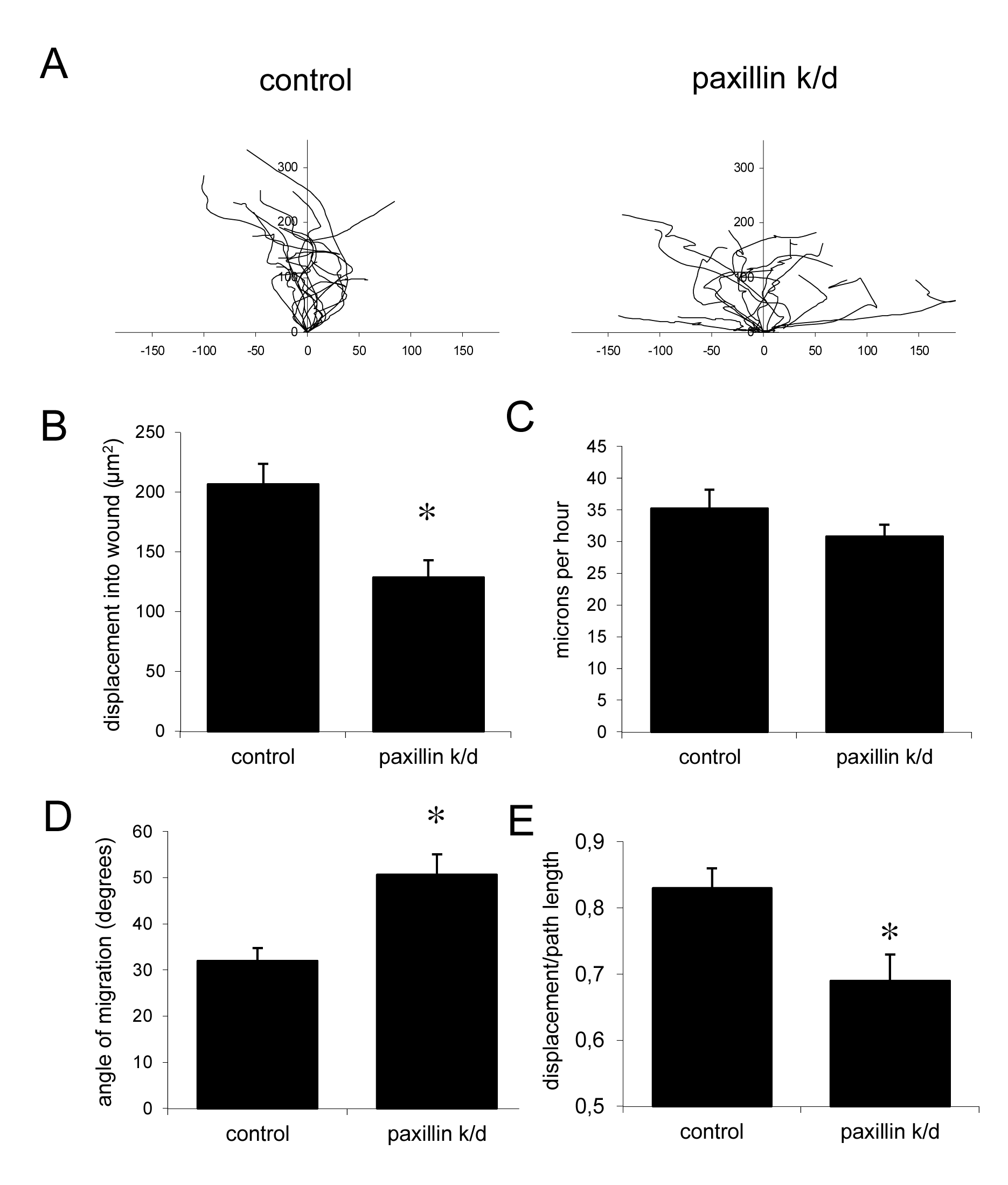

Supplement: Figure S5 — Knockdown of paxillin in human dermal fibroblasts leads to loss of directional persistence but not migration speed. A) Paths of individual cells at scrape wound edges over 6 h. B) Displacement into wounds. C) Migration speed. D) Average angle of migration. E) Displacement/path length, i.e. directional persistence. * p<0.001 compared to control. (TIF) [file pone.0028303.s005.tif]

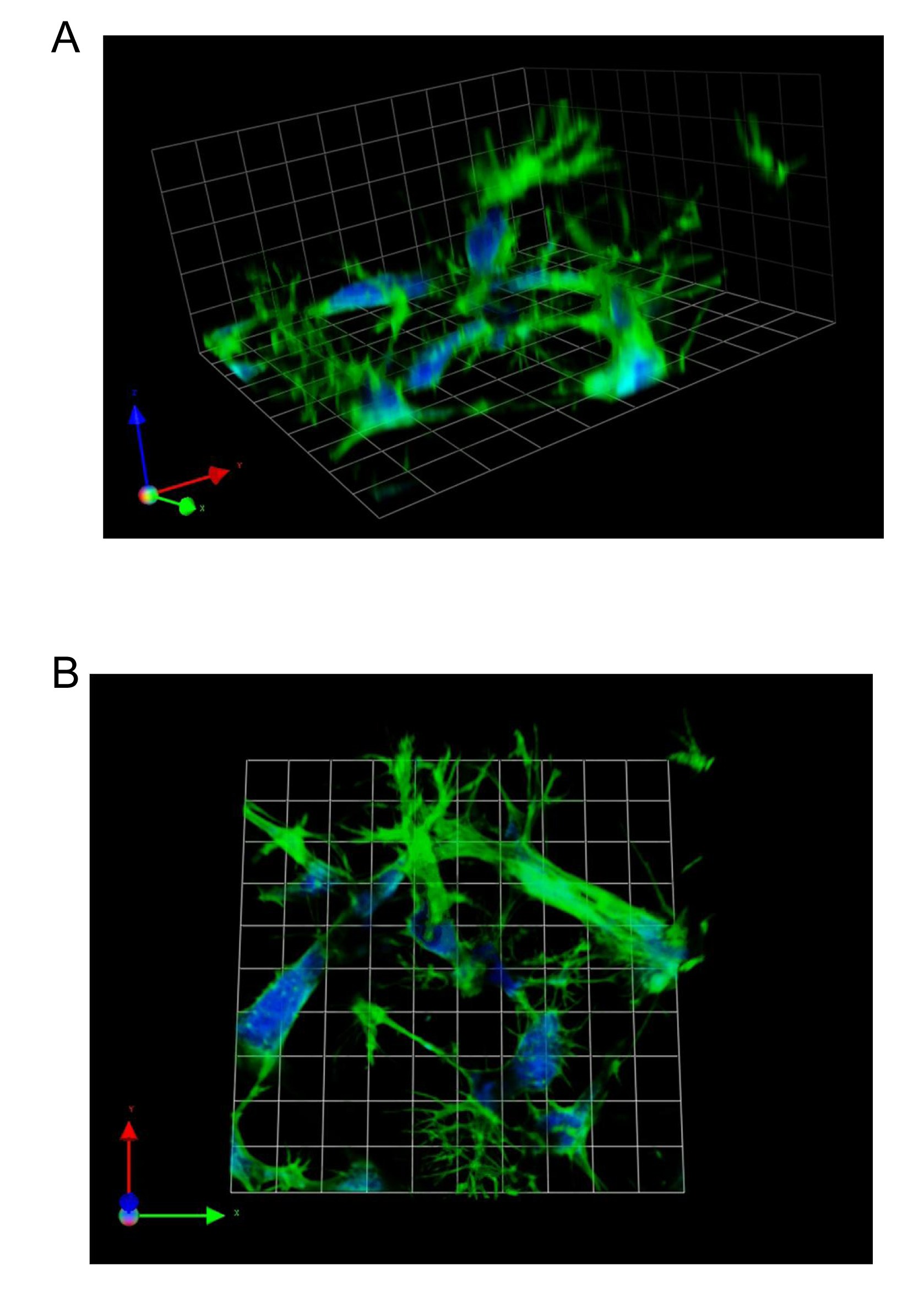

Supplement: Figure S6 — 3D reconstruction of invasive pax−/− cells in Matrigel. A) side-view of stack. B) Top-view of stack. Actin is labeled in green, DNA in blue. Length of grid unit = 12 µm. (TIF) [file pone.0028303.s006.tif]
